# Effects of Water Temperature, Stocking Density, and Exposure Period on the Susceptibility of Whiteleg Shrimp (*Penaeus vannamei*) to Waterborne White Spot Syndrome Virus

**DOI:** 10.3390/ani16182855

**Published:** 2026-09-10

**Authors:** Min Jae Kim, Jae-Ok Kim, Hee-Jung Choi, Mun-Gyeong Kwon, Chan-Il Park, Do-Hyung Kim, Kwang-Il Kim

**Affiliations:** 1 Aquatic Disease Control Division, National Fishery Products Quality Management Service (NFQS), Busan 49111, Republic of Korea; kimmj96@korea.kr (M.J.K.); kimjaeok@korea.kr (J.-O.K.); cchj77@korea.kr (H.-J.C.); mgkwon@korea.kr (M.-G.K.); 2 Department of Marine Biology and Aquaculture, College of Marine Science, Gyeongsang National University, Tongyeong 53064, Republic of Korea; parkci@gnu.ac.kr; 3 Department of Aquatic Life Medicine, Pukyong National University, Busan 48513, Republic of Korea; dhkim@pknu.ac.kr

**Keywords:** white spot syndrome virus, stocking density, water temperature, waterborne transmission, minimum infective dose

## Abstract

In pond-based shrimp culture, seawater exchange may introduce white spot syndrome virus (WSSV) from coastal waters, but infection risk at a given seawater viral concentration may vary with temperature, stocking density, and exposure duration. This study evaluated waterborne WSSV susceptibility in whiteleg shrimp (*Penaeus vannamei*) under these conditions. Shrimp were reared at 25 or 30 °C and at low (70), medium (140), or high (210 shrimp/m^2^) stocking densities; physiological responses were assessed, followed by short-term and continuous immersion challenges and a cohabitation challenge. Higher stocking-density conditions were associated with stress-related physiological changes and greater WSSV susceptibility. Under short-term exposure, 10^7^ WSSV genome copies/L caused infection only in the high-density group at 25 °C. Under continuous exposure, the minimum infective dose (MID) values were 10^5^ and 10^4^ WSSV genome copies/L at low and high stocking densities at 25 °C, and 10^6^ and 10^5^ WSSV genome copies/L, respectively, at 30 °C. Cohabitation transmission occurred at seawater viral loads below those associated with short-term immersion infection. By linking seawater viral concentrations with experimentally defined MID values and cohabitation-transmission outcomes, these results indicate that waterborne WSSV transmission risk should be assessed in relation to viral concentration, exposure duration, water temperature, and stocking-density conditions.

## Simple Summary

White spot syndrome virus causes severe disease and major losses in farmed shrimp, and it can enter ponds through seawater used for water exchange. However, the risk of infection may change with water temperature, crowding, and how long shrimp are exposed to contaminated water. We examined whiteleg shrimp kept at 25 or 30 °C and at three stocking densities, ranging from 70 to 210 shrimp per square meter. Shrimp were exposed to different virus concentrations for short or prolonged periods, and transmission from infected to healthy shrimp sharing the same water was also examined. Shrimp kept at higher densities showed greater stress-related changes and were generally more susceptible to infection. Infection occurred more readily at 25 °C than at 30 °C, and prolonged exposure allowed transmission at lower virus concentrations than short-term exposure. These findings show that the risk from incoming seawater cannot be judged from virus concentration alone. Considering temperature, stocking density, and exposure time together with seawater virus monitoring may help shrimp farmers manage water exchange more safely and reduce the risk of disease outbreaks.

## 1. Introduction

White spot syndrome virus (WSSV), the etiological agent of white spot disease (WSD), ranks among the most consequential viral pathogens in penaeid shrimp aquaculture worldwide [1,2,3]. Since its emergence in Asia, WSSV has spread to major shrimp-farming regions globally, causing substantial economic losses to the aquaculture industry [4,5,6]. Infected shrimp typically exhibit lethargy, reddish discoloration, and white spots on the exoskeleton; severe outbreaks commonly result in mass mortality within 10 days of infection [7,8]. WSD occurrence is strongly influenced by environmental conditions in shrimp ponds, and water temperature is regarded as one of the major factors governing disease outbreaks [9,10,11]. Water temperature may affect both the physiological status of shrimp and WSSV replication [3,12,13]. Previous studies have indicated that the optimal temperature range for WSSV replication is 23–29 °C [14,15]. Accordingly, WSD outbreaks have been reported mainly during the pre- and post-monsoon periods in Southeast Asia, when water temperatures fluctuate within the range favorable for WSSV propagation [16,17].

Whiteleg shrimp (*Penaeus vannamei*) farming has expanded worldwide, progressively shifting from extensive pond culture to more intensive production systems [18]. Although such intensification has contributed to increased productivity, it may also impair shrimp performance and compromise the rearing environment if not properly managed [19,20,21]. Intensive culture conditions may exacerbate disease outcomes by altering stress-related physiological status and increasing opportunities for pathogen transmission among shrimp [22,23,24]. Consistent with this, WSD occurrence and disease progression have been associated with stocking density in shrimp farms, and field investigations have identified stocking density as a risk factor for WSD occurrence [17,25]. Previous studies have examined the effects of stocking density on shrimp physiological responses, WSD progression, viral shedding, and interaction variability among infected shrimp [24,26,27]. However, how stocking density modifies WSSV infection risk following exposure to WSSV-contaminated rearing water remains insufficiently defined, particularly in the context of waterborne viral introduction.

Because waterborne transmission represents one of the principal routes of WSSV spread, assessing transmission risk via this route is essential for preventing disease outbreaks [28,29]. Several studies have investigated waterborne WSSV transmission risk using immersion challenges at different viral loads and shrimp life stages [30,31]. However, pond-based shrimp culture involves environmental and husbandry conditions that may alter the outcome of waterborne exposure, including seasonal fluctuations in water temperature and differences in stocking density [3,17,24]. In addition, WSSV may retain viability for prolonged periods through complex interactions with vectors or reservoirs in pond culture systems, thereby increasing the likelihood of repeated or sustained exposure to waterborne virus [32,33,34]. A previous study demonstrated that WSSV exposure duration can alter the minimum infective dose (MID) [15]. In that study, infection occurred at relatively low doses when the exposure period was extended, even when such doses were insufficient to induce infection under short-term exposure. Nevertheless, quantitative information remains limited regarding how water temperature, stocking density, and exposure duration jointly modify the waterborne MID and infection outcome of WSSV. Their combined effects on subsequent transmission risk under controlled, pond-relevant exposure scenarios also remain insufficiently defined.

Several previous studies have developed methods for concentrating viral particles from environmental water to quantify viral loads and investigate waterborne or horizontal transmission [35,36,37]. Kawato et al. [38] demonstrated that integrating field-based seawater viral-load monitoring with laboratory immersion challenges can provide a quantitative framework for assessing waterborne transmission risk. Similarly, an iron flocculation assay has been optimized for concentrating WSSV particles in seawater and shown to be applicable for monitoring WSSV in shrimp pond water [33]. However, for WSSV, seawater viral-load data alone are difficult to interpret in terms of infection risk unless the measured viral concentrations are linked to experimentally defined infective doses under relevant rearing conditions [33,38]. In shrimp pond culture, seawater exchange may represent a major biosecurity risk factor for the introduction of pathogens from external sources [11,39]. Because water exchange is generally conducted intermittently at defined intervals under controlled management [40], the introduction of WSSV-contaminated water may represent a repeated exposure event in pond culture systems. Therefore, experimentally defining how such periodic viral introduction leads to infection under pond-relevant temperature, stocking-density, and exposure-duration conditions is necessary for risk-based water-exchange management.

To address these gaps, the present study investigated how water temperature, stocking density, and exposure duration affect the susceptibility of whiteleg shrimp to waterborne WSSV. Particular emphasis was placed on defining variations in the waterborne MID under controlled exposure conditions relevant to pond-based shrimp culture. To determine whether stocking density affected the physiological status of shrimp before viral challenge, time-course changes in total hemocyte count, antioxidant-related markers, and stress-response proteins were analyzed under different stocking density conditions. Immersion challenges were then conducted at different viral doses and exposure durations to evaluate mortality patterns and temporal changes in viral loads in shrimp and seawater. In addition, cohabitation challenge models were used to assess whether sustained waterborne exposure could result in transmission at viral concentrations that were insufficient to induce infection under short-term exposure conditions. By integrating these experimental approaches, this study aimed to link WSSV concentrations in rearing water with infection outcomes, waterborne MID values, and subsequent transmission under pond-relevant temperature and stocking-density conditions. This approach provides a quantitative experimental basis for assessing WSSV transmission risk associated with the potential introduction of WSSV via coastal water used for water exchange in pond-based shrimp culture systems.

## 2. Materials and Methods

### 2.1. Experimental Shrimp

Whiteleg shrimp (*Penaeus vannamei*; 4.05 ± 0.96 g) used in this study were obtained from an aquaculture farm in Geoje, Republic of Korea. The WSSV-free status of the shrimp was confirmed by real-time polymerase chain reaction (PCR) using nine randomly selected individuals, as described in Section 2.3 of this manuscript. Before the experiments, the shrimp were acclimated for 2 weeks in ultraviolet (UV)-treated seawater at 25 ± 0.5 °C in a 250-L static tank at a stocking density of 70 shrimp/m^2^. During acclimation, 100% of the rearing water was exchanged daily, and the shrimp were fed a commercial diet (2.8–3.3 mm diameter; Dong-A, Busan, Republic of Korea) two times daily.

### 2.2. Virus

The virus used in this study was originally isolated from diseased whiteleg shrimp collected in Taean, Republic of Korea, in 2014 and was previously characterized as the WSSV-Te-14 isolate [33]. For inoculum preparation, muscle, pleopod, and gill tissues from WSSV-infected shrimp were homogenized in phosphate-buffered saline (PBS; Gibco, Grand Island, NY, USA) at a ratio of 1:20 (*w*/*v*). The homogenate was centrifuged at 3000× *g* for 30 min, and the resulting supernatant was filtered through a 0.45-µm syringe filter (Sartorius, Göttingen, Germany). The virus inoculum for the experimental infection was prepared within 7 days before each experiment. After quantitative analysis, as described in Section 2.3, the inoculum was stored at −80 °C until use.

### 2.3. Quantitative Analysis

To determine the WSSV genome copy number in whiteleg shrimp, total DNA was extracted from 10 mg of pleopod tissue using the yes^TM^ Cell Tissue Mini Kit (GenesGen, Busan, Republic of Korea), according to the manufacturer’s instructions. The extracted DNA was analyzed using real-time PCR according to the protocol described by Durand and Lightner [30]. Each 20 μL reaction mixture contained 10 μL of 2× HS Prime qPCR Premix (GeNet Bio, Daejeon, Republic of Korea), 300 nM each of the forward and reverse primers (0.6 μL each), 150 nM TaqMan probe (0.3 μL), 0.4 μL of 50× ROX dye, 7.1 μL of DEPC-treated water, and 1 μL of template DNA. Amplification was performed using a StepOne Real-Time PCR System (Applied Biosystems, Waltham, MA, USA). To ensure quantitative reproducibility, pDNA containing the WSSV *VP*664 gene (1461 bp) at three concentrations (10^6^, 10^4^, and 10^2^ copies/μL) was included in each run as a positive control. Quantitative values below the cut-off defined by the 95% limit of detection (4.67 WSSV genome copies/μL) were excluded from the analysis, as described previously [41]. The primer sets used in this study are listed in Table S1. The number of viral genome copies per milligram of shrimp tissue was calculated using Equation (1).

(1) $$WSSV\;genome\;copies/mg=\frac{\left(WSSV\;genome\;copies/\mu L\;of\;total\;DNA\right)\times \left(vol.\;of\;eluted\;total\;DNA\right)}{weight\;of\;tissues\;used\;to\;extract\;total\;DNA\;(mg)}$$

### 2.4. Iron Flocculation Assay for WSSV Detection in Seawater

WSSV particles in the rearing seawater were concentrated using the iron flocculation-direct elution (I·D) method, as described previously [42]. Briefly, seawater samples were sequentially filtered under vacuum through a glass microfiber filter (1.6 µm pore size; Whatman, Little Chalfont, UK) and an MF-Millipore membrane filter (0.45 µm pore size; Merck, Darmstadt, Germany). Subsequently, 50 µL of FeCl_3_ solution (4.83 g FeCl_3_·6H_2_O dissolved in 100 mL of distilled water; final concentration of 4.83 µg/mL in seawater) was added to 500 mL of pre-filtered seawater. The mixture was stirred using a magnetic stirrer (<120 rpm; Sigma-Aldrich, St. Louis, MO, USA) for 1 h at room temperature (25 °C). The resulting Fe-WSSV flocculates were collected on a polycarbonate membrane (1.0 µm pore size; Whatman, UK) using a peristaltic pump (<15 psi; Eyela, Tokyo, Japan). The membrane was then transferred to a 2-mL tube for total DNA extraction and quantitative analysis, as described in Section 2.3. The number of viral genome copies per liter of seawater was calculated using Equation (2).

(2) $$WSSV\;genome\;copies/L=\frac{\left(WSSV\;genome\;copies\;per\;\mu L\;of\;total\;DNA\right)\times \left(vol.\;of\;eluted\;total\;DNA\right)}{vol.\;of\;seawater\;used\;in\;iron\;flocculation\;assay\left(L\right)}$$

### 2.5. Experimental Design

#### 2.5.1. Experimental Environmental and Husbandry Conditions

To investigate the effects of water temperature and stocking density on WSSV susceptibility in whiteleg shrimp, experimental challenge trials were conducted under two temperature conditions and three stocking densities, based on previous studies [24,43]. The temperatures of 25 °C and 30 °C were selected because both values fall within the range of water temperatures observed in practical whiteleg shrimp pond or rearing systems during the grow-out period [33,44]. In addition, these temperatures represent contrasting WSD-risk conditions, with 25 °C corresponding to a temperature range favorable for WSSV propagation and 30 °C representing a warmer culture condition, as described previously [24]. The water quality of the exchange water was monitored using a ProDSS Multiparameter Digital Water Quality Meter (YSI, Yellow Springs, OH, USA). The exchange water for the 25 °C groups was maintained within the following ranges: temperature, 25 ± 0.5 °C; dissolved oxygen, 7.2 ± 1.0 mg/L; salinity, 32.1 ± 0.7‰; pH, 7.9 ± 0.5; and ammonia nitrogen (NH_3_-N), 12.4 ± 1.8 µg/L. The exchange water for the 30 °C groups was maintained within the following ranges: temperature, 30 ± 0.5 °C; dissolved oxygen, 6.7 ± 0.8 mg/L; salinity, 32.5 ± 1.0‰; pH, 7.8 ± 0.7; and ammonia nitrogen (NH_3_-N), 16.8 ± 2.1 µg/L.

Stocking density conditions were set as follows: low density, 70 shrimp/m^2^ (recommended condition); medium density, 140 shrimp/m^2^ (twice the recommended density); and high density, 210 shrimp/m^2^ (three times the recommended density) [43]. All three density levels are within the FAO-reported range for intensive *P. vannamei* pond stocking, approximately 60–300 PL/m^2^ [45], and were used to represent a gradient from the recommended Korean farming density to intensified pond-culture conditions [43]. During the experimental period, stocking density was maintained using a portable grid to account for cumulative mortality over time.

#### 2.5.2. Classification of WSSV Severity Grades in Whiteleg Shrimp

WSSV severity grades in whiteleg shrimp were classified based on viral load criteria established previously [41]. Briefly, shrimp were assigned to five severity grades according to viral load (WSSV genome copies/mg): G0, 10^1.67^–10^2.87^; G1, 10^2.87^–10^3.99^; G2, 10^3.99^–10^5.15^; G3, 10^5.15^–10^7.18^; and G4, >10^7.18^. The severity grade of each shrimp was determined based on the mean value obtained from duplicate tests. The correlation between WSSV severity grade and viral load is shown in Table S2.

#### 2.5.3. Criteria for WSSV Infection Confirmation and MID Determination

For MID determination, WSSV infection was confirmed primarily based on virological detection in shrimp tissue. Individual shrimp were classified as WSSV-positive when the WSSV genome copy number in pleopod tissue was equal to or greater than the 95% real-time PCR limit of detection described in Section 2.3. At the dose-group level, infection was considered confirmed when WSSV-positive shrimp were detected during time-course sampling. WSD-related mortality during the 14-day observation period was used as a supporting disease-related endpoint. Infection was not considered confirmed when WSSV was not detected above the real-time PCR cut-off in sampled shrimp and no WSD-related mortality was observed.

The waterborne MID was defined as the lowest tested WSSV concentration, expressed as WSSV genome copies/L, that produced confirmed infection under each experimental condition. MID assignment was based on the tested 10-fold serial dilution series, with reference to the absence of confirmed infection at the next lower tested dose.

### 2.6. Effect of Stocking Density on the Physiological Status of Whiteleg Shrimp

In Experiment 1 (Exp. 1), total hemocyte count (THC) and the relative expression levels of superoxide dismutase (*SOD*), catalase (*CAT*), and heat shock protein 70 (*HSP70*) were evaluated to assess the physiological responses of whiteleg shrimp to different stocking densities. The low- and medium-density groups each comprised 32 shrimp, whereas the high-density group comprised 42 shrimp. The low-density group was reared in a static water tank with dimensions of 80 cm × 60 cm × 30 cm, whereas the medium- and high-density groups were reared in static water tanks with dimensions of 50 cm × 40 cm × 30 cm at 25 ± 0.5 °C. The actual stocking densities in the low-, medium-, and high-density groups were 66.7, 160, and 210 shrimp/m^2^, respectively. Throughout the experimental period, the water depth was maintained at 0.25 m in all tanks, rearing water was completely replaced daily, and shrimp were fed a commercial diet twice daily.

Before the start of the stocking density experiment, hemolymph was collected from five randomly selected shrimp maintained in the acclimation tank and used as the 0 h control. Thereafter, hemolymph was collected from five shrimp per group on days 7, 14, 21, and 28 and used for THC determination and gene expression analyses. To minimize variation associated with molting status, shrimp showing signs of recent or imminent molting, including soft-shell condition or abnormal exoskeletal appearance, were excluded, and only shrimp with a normal exoskeleton were selected for hemolymph collection. Briefly, 100 μL of hemolymph was withdrawn from each shrimp using a 1-mL syringe preloaded with 150 μL of Alsever’s solution (Sigma-Aldrich, St. Louis, MO, USA) for anticoagulation, following the non-lethal sampling procedure described by Kim et al. [46]. A 200-μL aliquot of the resulting hemolymph–Alsever’s solution mixture was used for RNA extraction. For THC determination, 20 μL of the mixture was diluted 1:1 with Rose Bengal solution (1 mg/mL; Sigma-Aldrich, USA), and 10-μL aliquots were counted in triplicate using a hemocytometer [47]. Total RNA was extracted from 200 μL of the hemolymph–Alsever’s solution mixture using a yes^TM^ Total RNA Extraction Kit (GenesGen, Republic of Korea), and cDNA was synthesized using a PrimeScript 1st Strand cDNA Synthesis Kit (Takara Bio, Inc., Shiga, Japan). Relative gene expression was analyzed by real-time PCR using a StepOne Real-Time PCR System in a final reaction volume of 20 μL containing 10 μL of 2× Prime Q-Master Mix (GeNetBio, Daejeon, Republic of Korea), 1 μL of each primer, 7 μL of distilled water, and 1 μL of cDNA. Thermal cycling included initial denaturation at 95 °C for 15 min, 40 cycles of 95 °C for 15 s and 60 °C for 30 s, and a final hold at 60 °C for 1 min. Relative expression levels of SOD, CAT, and HSP70 were normalized to β-actin and calculated using the 2^−ΔΔCt^ method [48], with the day-0 group used as the control. Primer sets used are listed in Table S1.

### 2.7. Assessment of WSSV Susceptibility in Whiteleg Shrimp Under Different Water Temperature and Stocking Density Conditions

#### 2.7.1. Immersion Challenge Experiments Under Different Water Temperature and Stocking Density Conditions

**Figure 1:**
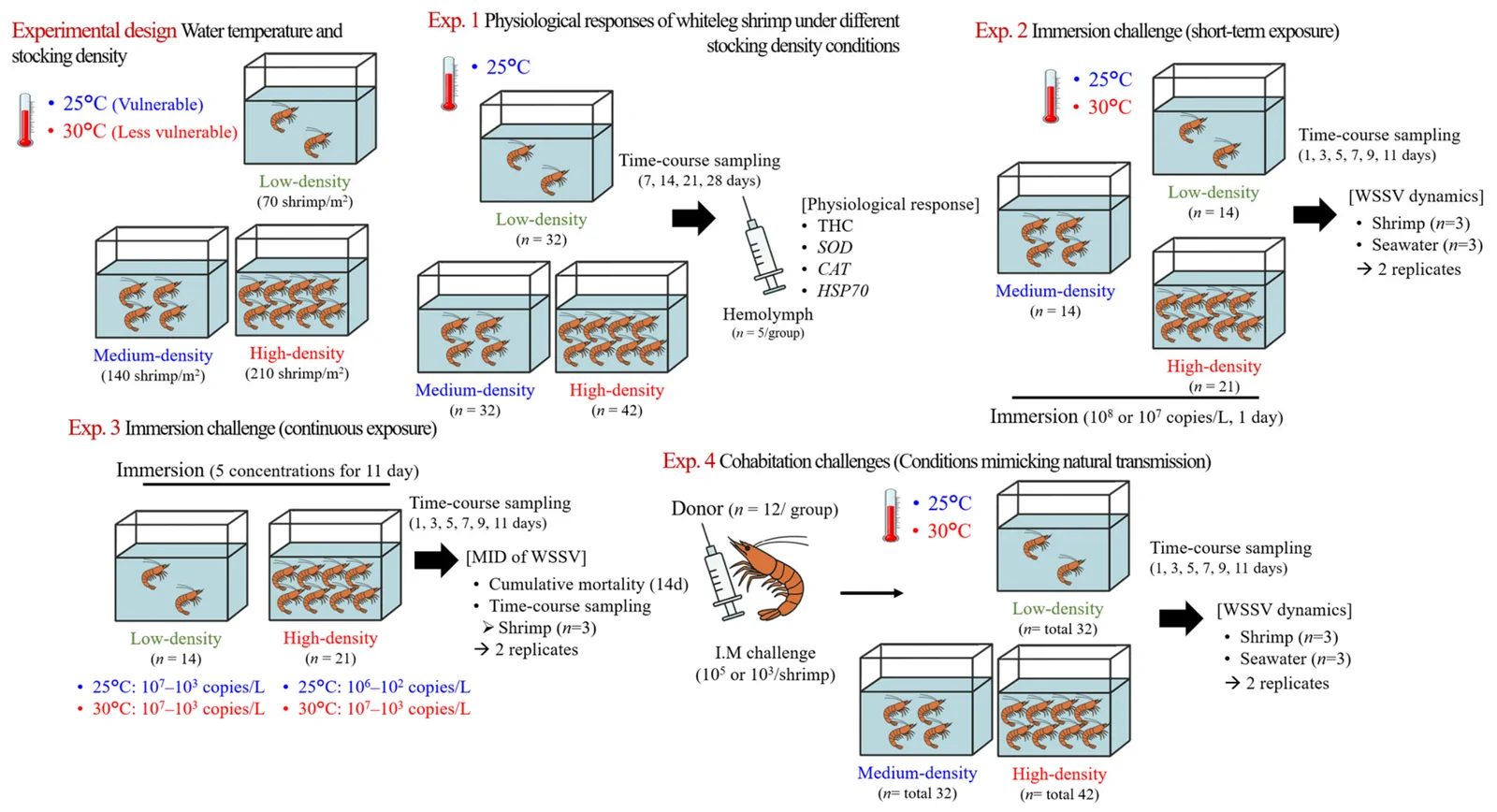
Schematic diagram of the experimental design used to investigate the effects of water temperature, stocking density, and exposure period on the susceptibility of whiteleg shrimp to waterborne white spot syndrome virus.

In Exps. 2 and 3, immersion challenge experiments were conducted to investigate WSSV susceptibility in whiteleg shrimp under different water temperature and stocking density conditions. Exp. 2 was designed as a short-term exposure scenario, in which shrimp were immersed for 1 day and 50% of the rearing water was replaced daily. Exp. 3 was designed as a continuous exposure scenario, in which shrimp were exposed to relatively low viral concentrations maintained at initial levels throughout the 11-day period. The low- and medium-density groups each comprised 14 shrimp per tank, whereas the high-density group comprised 21 shrimp per tank. The low-density group was reared in a static water tank with dimensions of 50 cm × 40 cm × 40 cm, whereas the medium- and high-density groups were reared in static water tanks with dimensions of 50 cm × 20 cm × 40 cm. The actual stocking densities in the low-, medium-, and high-density groups were 70, 140, and 210 shrimp/m^2^, respectively. A schematic diagram of the challenge experiments is presented in Figure 1.

In Exp. 2, a total of 588 shrimp were allocated to the low-, medium-, and high-density groups and acclimated to UV-treated seawater at 25 or 30 °C for 2 weeks. The shrimp were then challenged by immersion in WSSV-Te-14 at two doses (10^8^ and 10^7^ WSSV genome copies/L). Shrimp immersed in PBS-spiked seawater under the same conditions served as negative controls. After immersion, cumulative mortality was monitored for 14 days using two replicate tanks per group, and the shrimp were fed a commercial diet twice daily. In addition, 500-mL rearing seawater samples were collected in triplicate before water replacement at 1, 3, 5, 7, 9, and 11 dpi to determine viral loads using the I·D method (Section 2.4). For time-course sampling, an additional 588 shrimp were assigned to separate groups under the same conditions, and three shrimp per group were sampled at 1, 3, 5, 7, 9, and 11 dpi for quantitative analysis (Section 2.3).

In Exp. 3, a total of 840 shrimp were allocated to the low- and high-density groups and acclimated to UV-treated seawater at 25 or 30 °C for 2 weeks. They were then challenged by immersion with WSSV-Te-14 at five 10-fold serially diluted doses selected based on the results of Exp. 2. At 25 °C, the low- and high-density groups were challenged at doses ranging from 10^7^ to 10^3^ and from 10^6^ to 10^2^ WSSV genome copies/L, respectively. At 30 °C, both the low- and high-density groups were challenged with doses ranging from 10^7^ to 10^3^ WSSV genome copies/L. Shrimp immersed in PBS-spiked seawater under the same conditions were used as negative controls. After immersion, cumulative mortality was monitored for 14 days using two replicate tanks per group, and the shrimp were fed a commercial diet twice daily. For time-course sampling, an additional 840 shrimp were assigned to separate groups under the same conditions, and three shrimp per group were sampled at 1, 3, 5, 7, 9, and 11 dpi for quantitative analysis (Section 2.3).

The WSSV immersion inoculum was prepared in two steps. First, 0.1 L of the viral suspension was diluted with 4.9 L of UV-sterilized seawater to prepare a 5-L stock solution. The stock solution was then further diluted with UV-sterilized seawater to obtain the final challenge dose in a total volume of 60 or 120 L. These volumes corresponded to the working volumes of the tanks, with the water depth maintained at 0.30 m in all tanks. For time-course sampling, only live shrimp were collected, with priority given to those exhibiting lethargy or moribundity. In Exp. 2, 50% of the rearing water was replaced daily with UV-sterilized seawater. In Exp. 3, 100% of the rearing water was replaced every 2 days with WSSV immersion inoculum at the initial challenge dose.

#### 2.7.2. Cohabitation Challenge Experiment Under Different Water Temperature and Stocking Density Conditions

In Exp. 4, a cohabitation challenge experiment was conducted to examine WSSV infection dynamics in whiteleg shrimp under different water temperature and stocking density conditions mimicking natural infection. The low- and medium-density groups each consisted of 32 shrimp, whereas the high-density group consisted of 42 shrimp. The low-density group was reared in a static water tank with dimensions of 80 cm × 60 cm × 30 cm, whereas the medium- and high-density groups were reared in static water tanks with dimensions of 50 cm × 40 cm × 30 cm at 25 ± 0.5 °C. The actual stocking densities in the low-, medium-, and high-density groups were 66.7, 160, and 210 shrimp/m^2^, respectively.

A total of 1272 shrimp were allocated to the low-, medium-, and high-density groups and acclimated to UV-treated seawater at 25 or 30 °C for 2 weeks. Thereafter, shrimp (*n* = 12 per group) were intramuscularly injected with 0.1 mL of diluted WSSV-Te-14 at two doses (10^5^ or 10^3^ WSSV genome copies/shrimp). As a negative control, shrimp (*n* = 12) were intramuscularly injected with the same volume (0.1 mL) of PBS. Following injection, plastic cages were installed in each tank to separate the experimentally infected shrimp (donors) from healthy shrimp (recipients). Cumulative mortality was monitored for 14 days using two replicate tanks per group. In addition, 500-mL rearing seawater samples were collected in triplicate before water replacement at 1, 3, 5, 7, 9, and 11 dpi to determine viral loads using the I·D method (Section 2.4). For time-course sampling, an additional 1272 shrimp were assigned to separate groups under the same conditions, and three shrimp per group were sampled at 1, 3, 5, 7, 9, and 11 dpi for quantitative analysis (Section 2.3). During the experiment, 50% of the rearing water was replaced daily with UV-sterilized seawater, and the shrimp were fed a commercial diet twice daily.

### 2.8. Statistical Analysis

Significant differences in THC and relative expression levels of *SOD*, *CAT*, and *HSP70* in Exp. 1 were assessed using two-way analysis of variance (ANOVA), followed by Dunnett’s multiple comparison test against the control group. Prior to analysis, model assumptions were evaluated by inspecting Q-Q plots for residual normality and residual plots for homogeneity of variance and overall model adequacy (Figure S1). Mortality trends of recipient shrimp in the cohabitation challenge groups in Exp. 4 were evaluated using Kaplan–Meier survival curves, and significant differences were assessed using the log-rank test, followed by Holm’s pairwise comparison test. Survival analysis was performed using pooled data from groups with identical water temperature and stocking density conditions, regardless of the inoculation dose administered to the donors (10^5^ and 10^3^ WSSV genome copies/shrimp). The time to 50% mortality derived from the Kaplan–Meier curves was used only to describe the progression of recipient-shrimp mortality in the cohabitation challenge. Two-way ANOVA and log-rank tests were performed in R version 4.5.0 [49] using the ‘car’ and ‘survival’ packages.

## 3. Results

### 3.1. Physiological Responses of Whiteleg Shrimp Under Different Stocking Density Conditions

**Figure 2:**
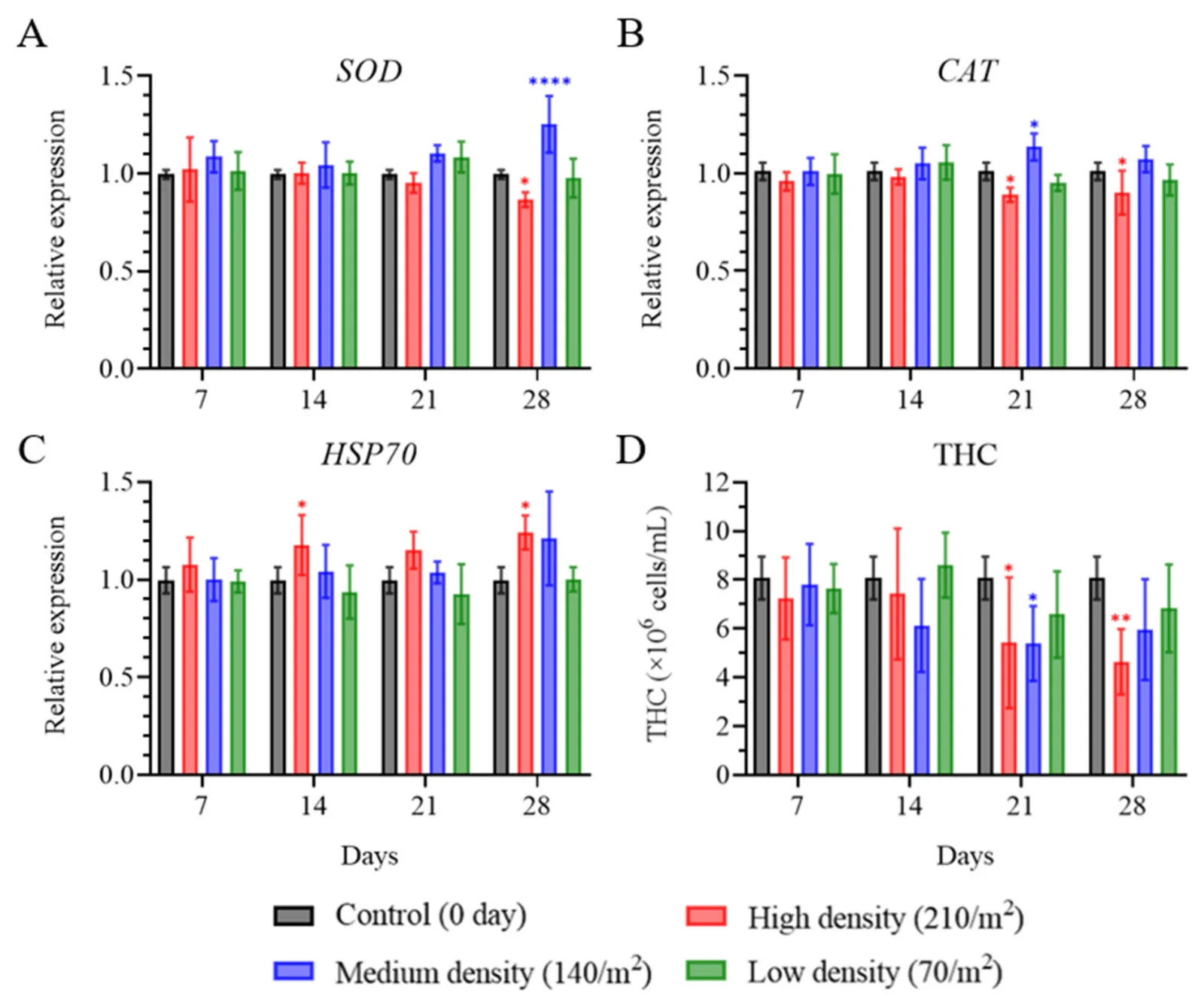
Physiological parameters of whiteleg shrimp under different stocking density conditions, including (**A**) *SOD*, (**B**) *CAT*, (**C**) *HSP70*, and (**D**) THC. Whiteleg shrimp were reared at three different stocking densities (low, 70; medium, 140; and high, 210 shrimp/m^2^) for 28 days. Hemolymph (100 µL) was collected from five shrimp per group on days 7, 14, 21, and 28 and used for THC determination and gene expression analyses. THC was determined using a hemocytometer. Relative expression levels of *SOD*, *CAT*, and *HSP70* were normalized to β-actin and calculated using the 2^–ΔΔCt^ method, with the day-0 group used as the control. Statistical significance was determined using two-way ANOVA followed by Dunnett’s multiple comparison test against the control group (*ns*, not significant; * *p* < 0.05; ** *p* < 0.01; *** *p* < 0.001; **** *p* < 0.0001). THC, total hemocyte count; *SOD*, superoxide dismutase; *CAT*, catalase; *HSP70*, heat shock protein 70; ANOVA, analysis of variance.

In Exp. 1, THC and the relative expression levels of *SOD*, *CAT*, and *HSP70* were evaluated to assess the physiological responses of whiteleg shrimp under different stocking density conditions. The 0 h control shrimp exhibited a THC of 8.08 ± 0.88 × 10^6^ cells/mL. In the low-density group, mean THC remained within approximately 7–9 × 10^6^ cells/mL throughout the 28-day experimental period and did not differ significantly from that of the control group (Figure 2). In the medium-density group, mean THC decreased significantly to 5.39 ± 1.54 × 10^6^ cells/mL on day 21. In the high-density group, mean THC was significantly reduced on days 21 and 28, reaching 5.43 ± 2.68 × 10^6^ and 4.64 ± 1.34 × 10^6^ cells/mL, respectively (Figure 2). No significant changes in *SOD*, *CAT*, or *HSP70* expression were observed in the low-density group, consistent with the THC results. In contrast, the medium-density group exhibited significantly increased *CAT* expression on day 21 and significantly increased *SOD* expression on day 28 (Figure 2). In the high-density group, *CAT* expression was significantly reduced on day 21, while both *SOD* and *CAT* expression were significantly reduced on day 28. In contrast, *HSP70* expression increased gradually and differed significantly from that of the control group on days 14 and 28 (Figure 2).

### 3.2. (Exp. 2) Susceptibility of Shrimp to WSSV Under Different Water Temperature and Stocking Density Conditions

#### 3.2.1. Cumulative Mortality of Shrimp at Different Immersion Doses

**Figure 3:**
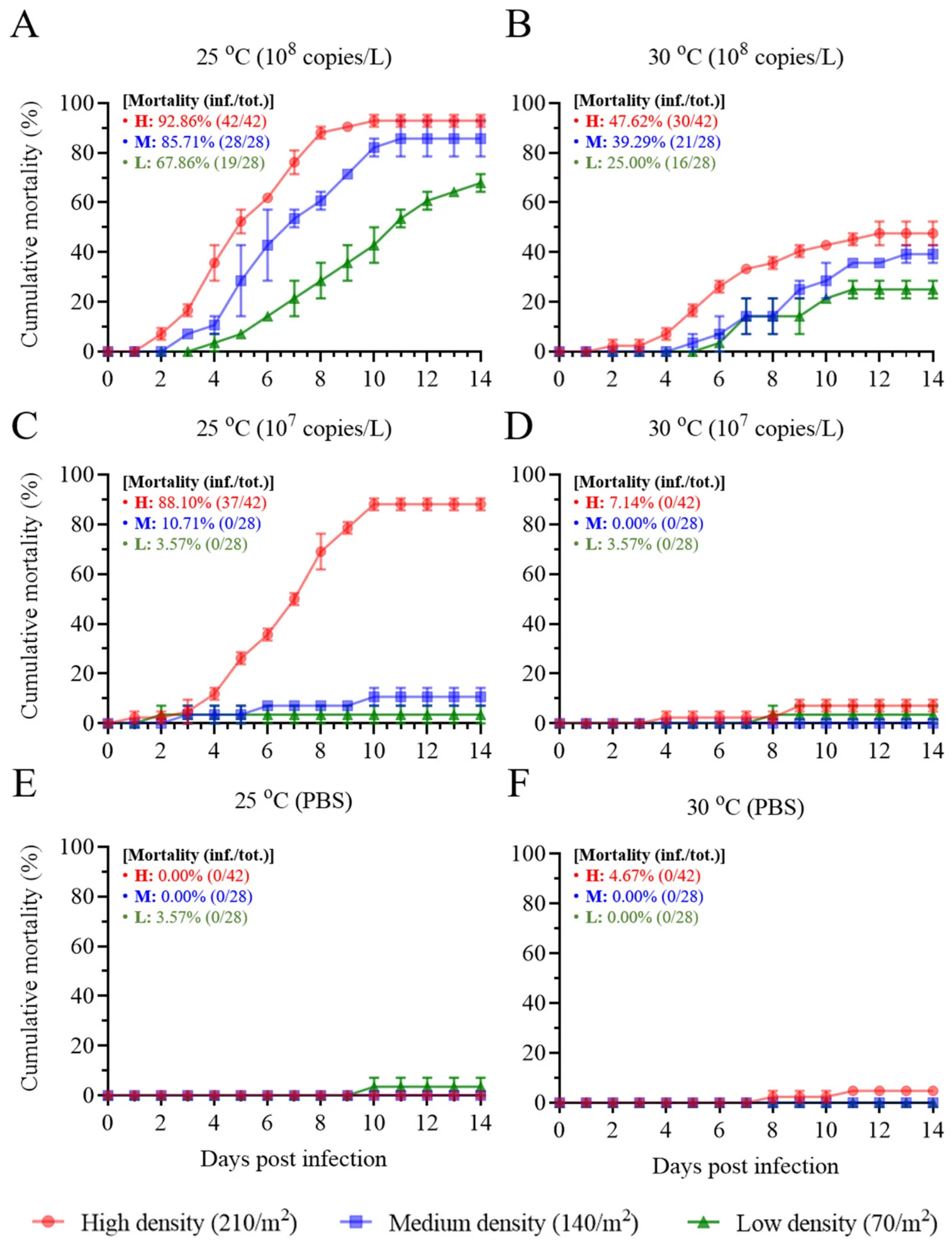
Cumulative mortality of whiteleg shrimp in Experiment 2 (short-term immersion exposure) during a 14-day observation period. Whiteleg shrimp (*Penaeus vannamei*; 4.05 ± 0.96 g) were assigned to low-, medium-, and high-density groups and acclimated at (**A**,**C**,**E**) 25 or (**B**,**D**,**F**) 30 °C for 2 weeks, after which they were challenged by immersion with WSSV-Te-14 at doses of (**A**,**B**) 10^8^ or (**C**,**D**) 10^7^ WSSV genome copies/L. (**E**,**F**) Shrimp immersed in PBS-spiked seawater under the same conditions were used as negative controls. Following immersion, 50% of the rearing water was replaced daily with UV-sterilized seawater, and the shrimp were fed a commercial diet twice daily. Cumulative mortality was monitored under each condition for 14 days, and duplicate tests were conducted. Error bars indicate the range of duplicate tests. WSSV, white spot syndrome virus; PBS, phosphate-buffered saline; UV, ultraviolet.

In Exp. 2, immersion challenges were conducted to evaluate the effects of water temperature (25 or 30 °C) and stocking density (low, medium, and high) on WSSV susceptibility in shrimp at two immersion doses (10^8^ and 10^7^ WSSV genome copies/L). Among shrimp exposed to 10^8^ WSSV genome copies/L, cumulative mortality increased from the low- to high-density groups at both temperatures (Figure 3). At 25 °C, mortality occurred earlier as stocking density increased, and final cumulative mortality ranged from 67.86% to 92.86%. At 30 °C, cumulative mortality was lower than at 25 °C but still increased from 25.00% in the low-density group to 47.62% in the high-density group (Figure 3). At 10^7^ WSSV genome copies/L, mortality was detected only in the high-density group at 25 °C, with a cumulative mortality of 88.10%. No mortality occurred in the other groups during the 14-day observation period (Figure 3).

#### 3.2.2. Time-Course Dynamics of WSSV in Seawater and Shrimp and WSSV Severity Grades

To further characterize the effects of water temperature and stocking density on WSSV susceptibility in Exp. 2, time-course changes in WSSV dynamics in seawater and shrimp were examined. Viral loads in seawater are presented as mean ± standard deviation, whereas viral loads in shrimp and WSSV severity grades are presented as medians (interquartile range). Summary values are presented in Table 1, and detailed time-course values are shown in Table S3.

**Figure 4:**
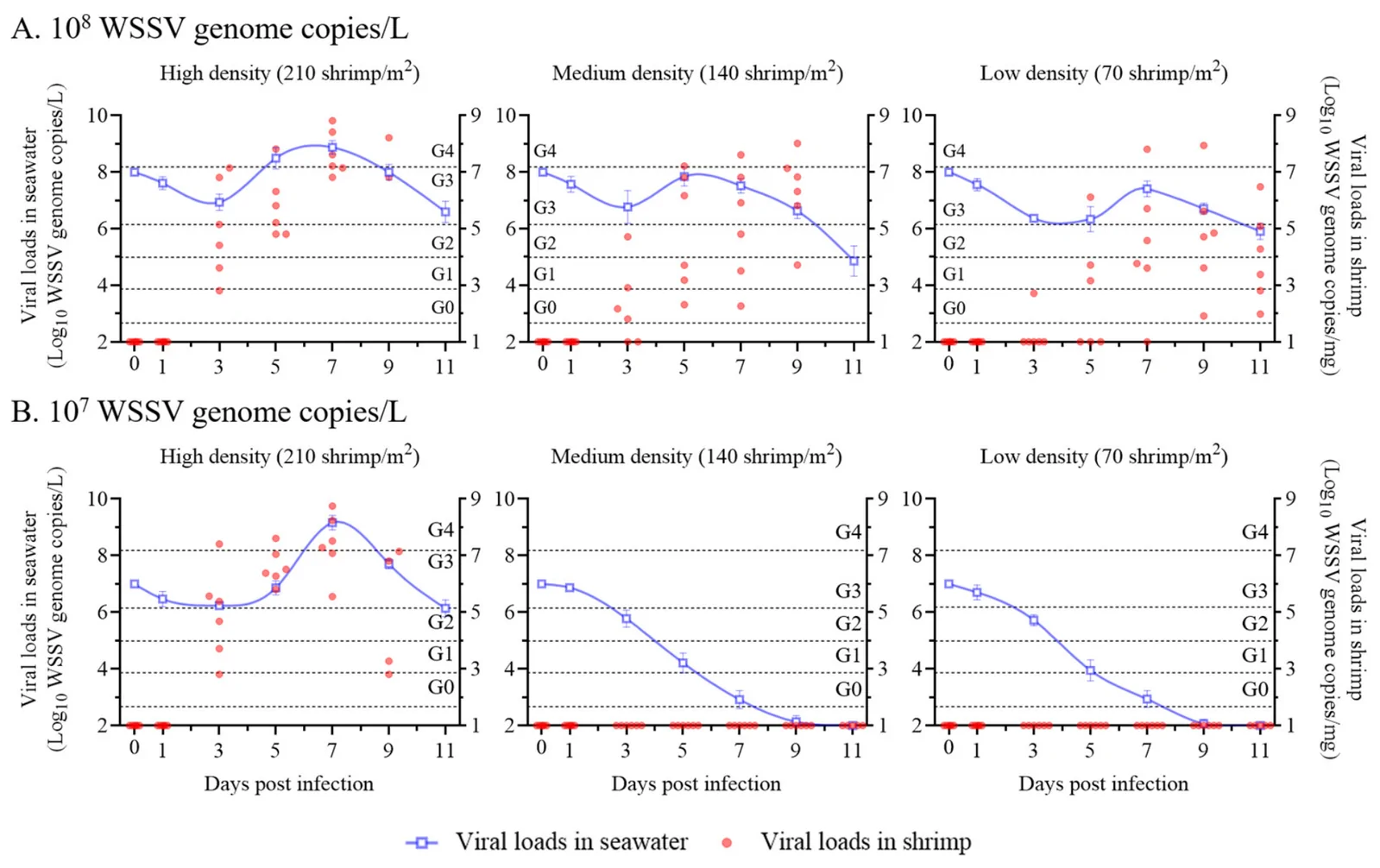
Time-course of viral loads in seawater and shrimp after immersion challenge with WSSV at concentrations of (**A**) 10^8^ and (**B**) 10^7^ WSSV genome copies/L at 25 °C in Experiment 2. Viral loads in seawater and shrimp (*n* = 3 per sampling day) were determined at 1, 3, 5, 7, 9, and 11 days post-infection using duplicate tests. Seawater viral loads were determined from 500-mL rearing seawater samples using the I·D method and are presented as log_10_ WSSV genome copies/L, whereas shrimp viral loads are presented as log_10_ WSSV genome copies/mg. WSSV severity grades were classified according to previously established viral load criteria for whiteleg shrimp [41], and horizontal dashed lines indicate the range of each disease severity grade. I·D, iron flocculation–direct elution; WSSV, white spot syndrome virus.

At 25 °C, in the groups exposed to 10^8^ WSSV genome copies/L, WSSV infection was first detected at 3 dpi, and median viral loads peaked at 9 dpi regardless of stocking density (Table 1). Shrimp viral loads increased earlier and progressed to higher WSSV severity grades in the higher-density groups. At 9 dpi, median viral loads increased from 10^4.78^ [10^4.01^–10^5.23^] WSSV genome copies/mg in the low-density group to 10^7.52^ [10^7.17^–10^7.87^] WSSV genome copies/mg in the high-density group, corresponding to G2–G3 and G3–G4 severity grades, respectively (Table 1). In seawater, viral loads decreased after immersion until 3 dpi, when WSSV infection was first detected in shrimp (Figure 4). At this time point, seawater viral loads ranged from 10^6.37±0.08^ to 10^6.94±0.28^ WSSV genome copies/L across the density groups. Thereafter, seawater viral loads increased until 7, 5, and 7 dpi in the low-, medium-, and high-density groups, respectively. Viral loads then decreased toward the end of the observation period (Table 1).

**Figure 5:**
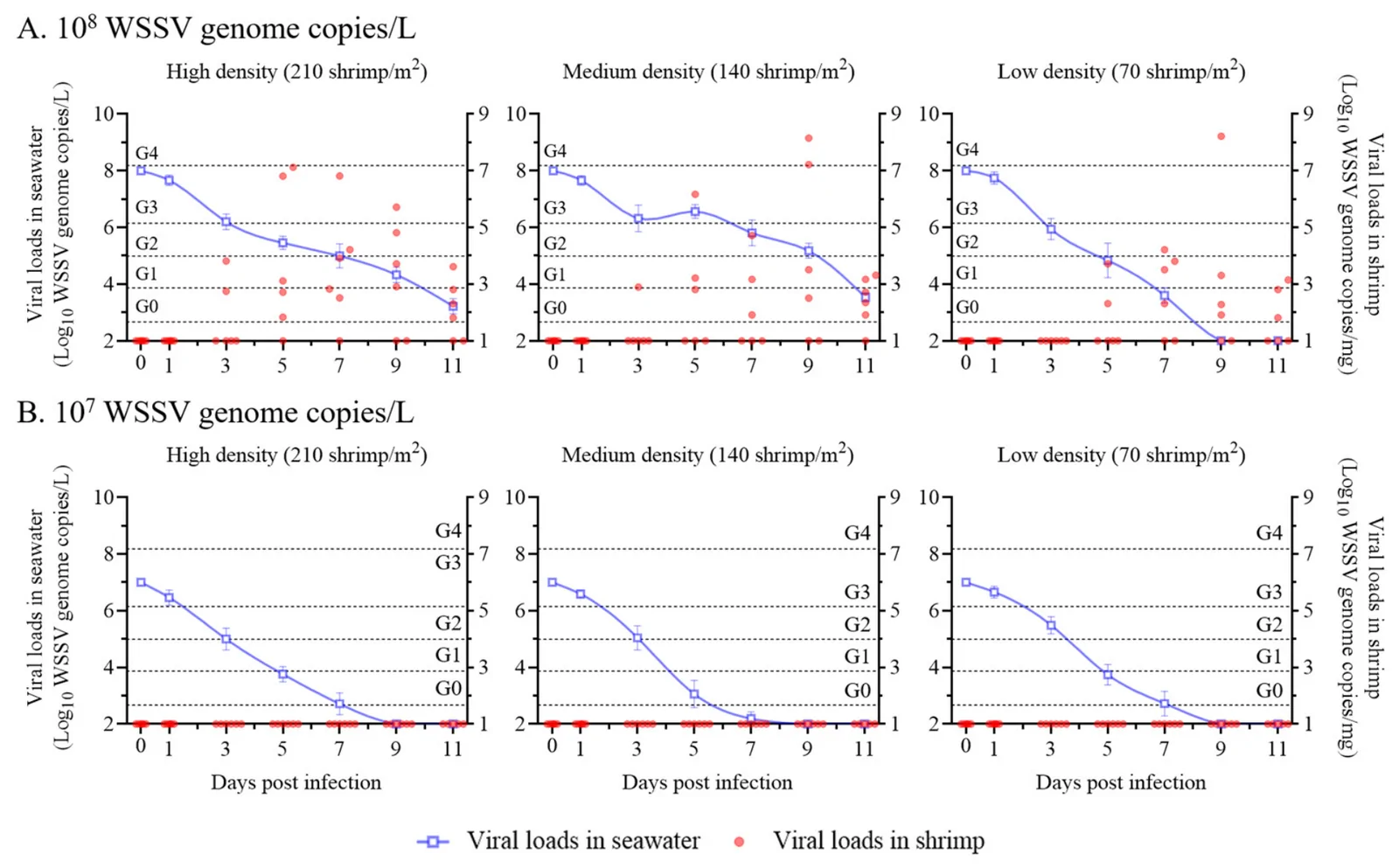
Time-course of viral loads in seawater and shrimp (*n* = 3 per sampling day) after immersion challenge with WSSV at concentrations of (**A**) 10^8^ and (**B**) 10^7^ WSSV genome copies/L at 30 °C in Experiment 2. WSSV severity grades were classified according to previously established viral load criteria for whiteleg shrimp [41], and horizontal dashed lines indicate the range of each disease severity grade. I·D, iron flocculation–direct elution; WSSV, white spot syndrome virus.

At 30 °C, in the groups exposed to 10^8^ WSSV genome copies/L, WSSV infection was first detected at 5 dpi in the low-density group, whereas it was first detected at 3 dpi in the medium- and high-density groups (Figure 5). In shrimp, viral loads remained at approximately 10^2^–10^3^ WSSV genome copies/mg in the low-density group until 11 dpi, whereas they peaked at 9 dpi in the high-density group, reaching 10^4.26^ [10^3.72^–10^4.81^] WSSV genome copies/mg (G1–G2) (Table 1). In seawater, viral loads decreased after immersion in all density groups. At the time when WSSV infection was first detected in shrimp, seawater viral loads were 10^4.84±0.61^ and 10^6.21±0.28^ WSSV genome copies/L in the low- and high-density groups, respectively. Thereafter, seawater viral loads continued to decrease, and WSSV was not detected at 9 and 11 dpi in the low-density group, whereas it remained detectable until 11 dpi in the medium- and high-density groups (Figure 5 and Table 1).

In the groups exposed to 10^7^ WSSV genome copies/L, WSSV infection was detected only in the high-density group at 25 °C. In this group, shrimp viral loads peaked at 7 dpi, reaching 10^7.40^ [10^7.18^–10^7.88^] WSSV genome copies/mg (G4) (Figure 4). In seawater, viral loads decreased to 10^6.24±0.09^ WSSV genome copies/L at 3 dpi. Thereafter, viral loads increased until 7 dpi, when shrimp viral loads also peaked, and then decreased toward the end of the observation period. In all other groups, WSSV was not confirmed in shrimp, and seawater viral loads declined to approximately 10^2^ WSSV genome copies/L by 7 or 9 dpi (Table 1).

### 3.3. (Exp. 3) MID of WSSV Under Continuous Exposure Conditions

In Exp. 3, the MID of WSSV was determined under continuous exposure conditions. Based on the infection-confirmation criteria described in Section 2.5.3, the MID was assigned as the lowest tested dose at which WSSV infection was confirmed, with no confirmed infection at the next lower tested dose.

**Figure 6:**
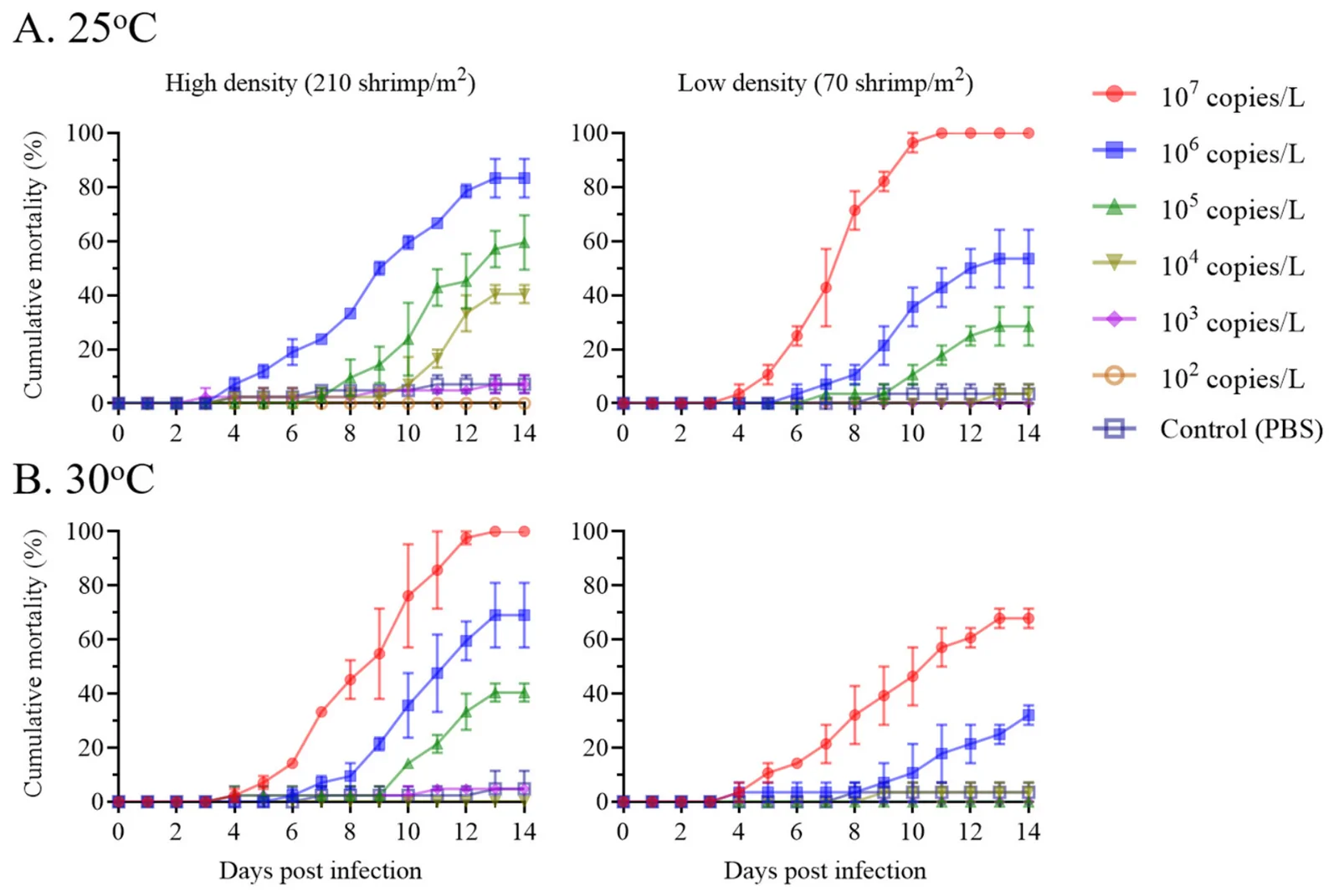
Cumulative mortality of whiteleg shrimp in Experiment 3 (continuous immersion exposure) during a 14-day observation period. Whiteleg shrimp were assigned to low- and high-density groups and acclimated at (**A**) 25 °C or (**B**) 30 °C for 2 weeks, after which they were challenged by immersion with WSSV-Te-14 at five 10-fold serially diluted doses selected based on the results of Experiment 2. Following immersion, the rearing water was replaced every 2 days with seawater spiked with WSSV at the initial challenge dose, and shrimp were fed a commercial diet twice daily. Cumulative mortality was monitored under each condition for 14 days, with duplicate tests conducted. Error bars indicate the range of the duplicate tests. WSSV, white spot syndrome virus.

At 25 °C, cumulative mortality decreased with decreasing exposure dose in both density groups. In the low-density group, cumulative mortality decreased from 100.00% at 10^7^ to 50.00% at 10^5^ WSSV genome copies/L, and the first detection of WSSV infection was delayed from 3 to 7 dpi (Table 2). In the high-density group, cumulative mortality decreased from 83.33% at 10^6^ to 40.48% at 10^4^ WSSV genome copies/L, and the first detection of infection was delayed from 3 to 9 dpi (Figure 6A). WSSV infection was not confirmed at 10^4^ and 10^3^ WSSV genome copies/L in the low- and high-density groups, respectively. Accordingly, the MID values at 25 °C were determined as 10^5^ and 10^4^ WSSV genome copies/L in the low- and high-density groups, respectively.

At 30 °C, mortality and WSSV detection showed a similar dose-related pattern, but infection was confirmed only at higher dose ranges than at 25 °C. In the low-density group, cumulative mortality decreased from 67.86% at 10^7^ to 32.14% at 10^6^ WSSV genome copies/L, and the first detection of WSSV infection was delayed from 3 to 7 dpi (Figure 6B). In the high-density group, cumulative mortality decreased from 100.00% at 10^7^ to 40.48% at 10^5^ WSSV genome copies/L, and the first detection of infection was delayed from 3 to 7 dpi (Table 2). WSSV infection was not confirmed at 10^5^ and 10^4^ WSSV genome copies/L in the low- and high-density groups, respectively. Accordingly, the MID values at 30 °C were determined as 10^6^ and 10^5^ WSSV genome copies/L in the low- and high-density groups, respectively. Detailed dose-specific cumulative mortality and WSSV detection results are shown in Table S4.

### 3.4. (Exp. 4) Cohabitation Challenge Under Different Water Temperature and Stocking Density Conditions

#### 3.4.1. Cumulative Mortality of Shrimp During Cohabitation Challenge

In Exp. 4, cohabitation challenges were conducted to evaluate WSSV susceptibility in shrimp under different water temperatures (25 and 30 °C) and stocking densities (low, medium, and high). Donor shrimp (*n* = 12 per group) were intramuscularly injected with WSSV-Te-14 at two doses (10^5^ or 10^3^ WSSV genome copies/shrimp), and waterborne transmission to recipient shrimp was evaluated while donors and recipients were housed in separate plastic cages in the same tank.

**Figure 7:**
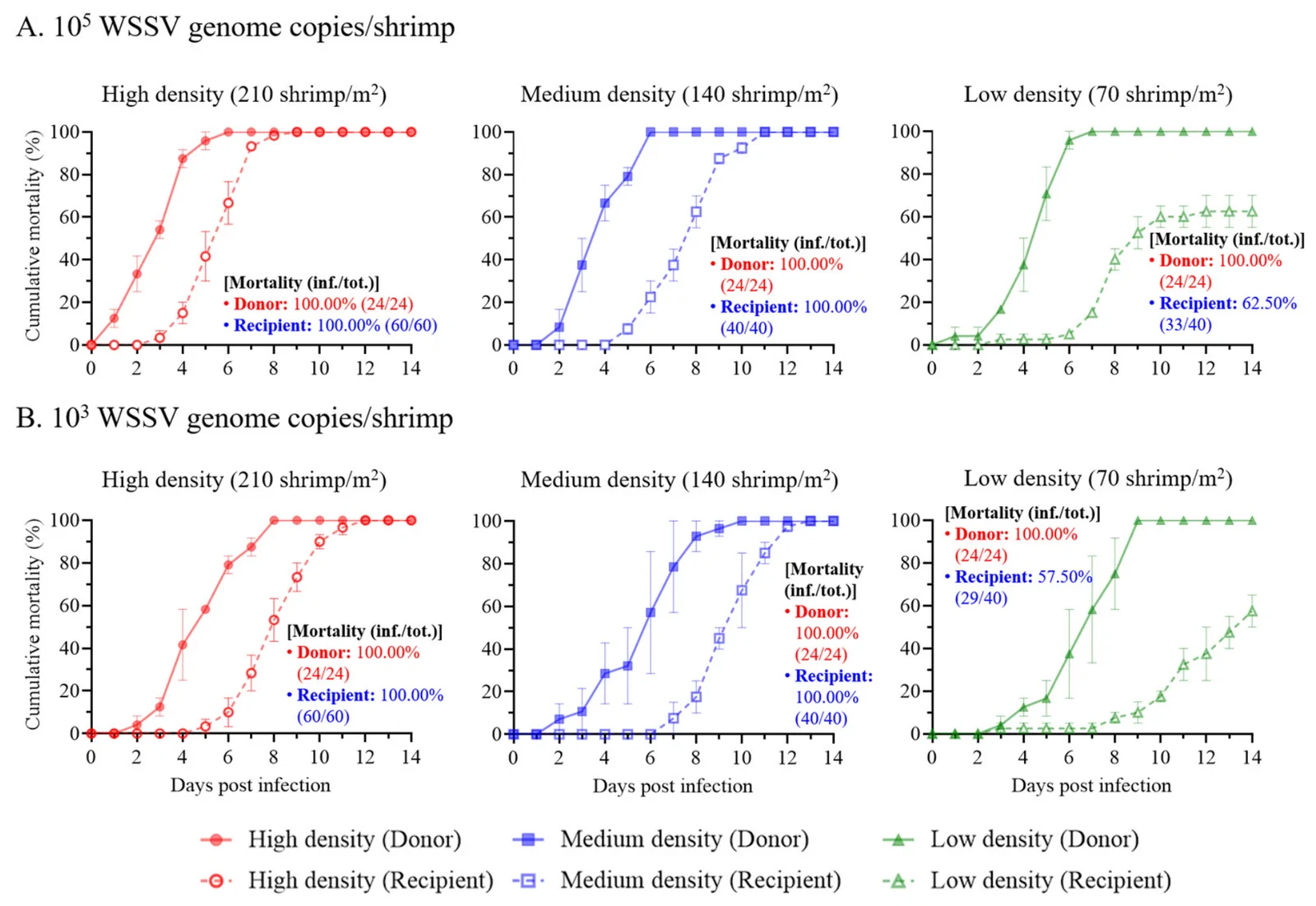
Cumulative mortality of whiteleg shrimp during the 14-day cohabitation challenge in Experiment 4 at 25 °C. Donor shrimp (*n* = 12 per group) were intramuscularly injected with 0.1 mL of diluted WSSV-Te-14 at doses of (**A**) 10^5^ or (**B**) 10^3^ WSSV genome copies/shrimp. Following injection, plastic cages were installed in each tank to separate infected donor shrimp from healthy recipient shrimp. During the experimental period, 50% of the rearing water was replaced daily with UV-sterilized seawater, and the shrimp were fed a commercial diet twice daily. Cumulative mortality was monitored under each condition for 14 days, and duplicate tests were conducted. Error bars indicate the range of duplicate tests. WSSV, white spot syndrome virus; UV, ultraviolet.

At 25 °C, donor shrimp exhibited 100% cumulative mortality, regardless of inoculation dose or stocking density. In recipient shrimp, mortality occurred earlier in the high-density group than in the low-density group (Figure 7). In groups inoculated with 10^5^ WSSV genome copies/shrimp, the first WSSV-associated mortality in recipient shrimp was observed at 6 dpi in the low-density group and at 3 dpi in the high-density group, and cumulative mortality increased from 62.50% to 100.00% (Figure 7). In groups inoculated with 10^3^ WSSV genome copies/shrimp, cumulative mortality of recipient shrimp increased from 57.50% in the low-density group to 100.00% in the high-density group (Figure 7).

**Figure 8:**
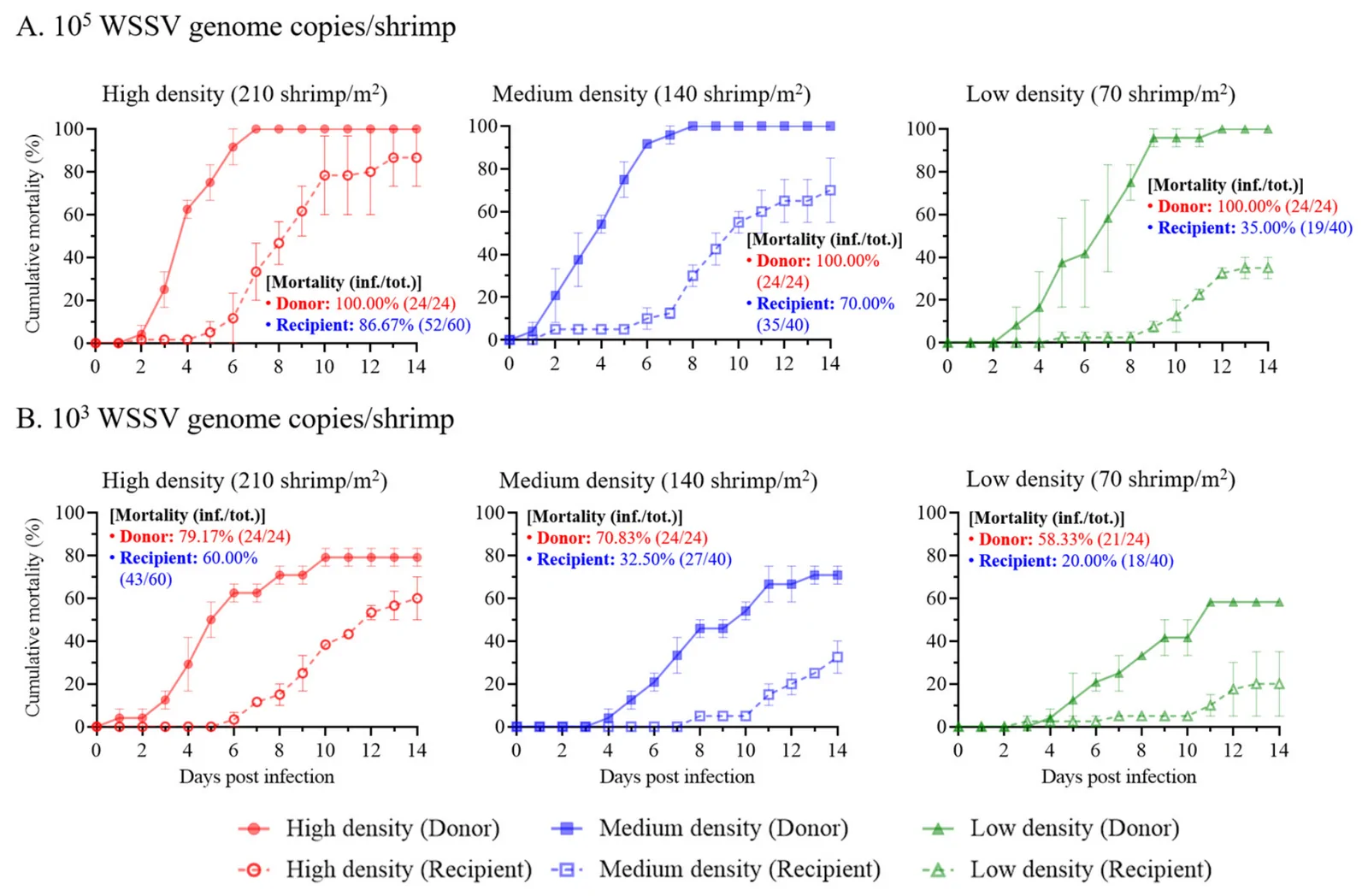
Cumulative mortality of whiteleg shrimp during the 14-day cohabitation challenge in Experiment 4 at 30 °C. Donor shrimp (*n* = 12 per group) were intramuscularly injected with 0.1 mL of diluted WSSV-Te-14 at doses of (**A**) 10^5^ or (**B**) 10^3^ WSSV genome copies/shrimp. Cumulative mortality was monitored under each condition for 14 days, and duplicate tests were conducted. Error bars indicate the range of duplicate tests. WSSV, white spot syndrome virus; UV, ultraviolet.

At 30 °C, both the timing and cumulative mortality of recipient shrimp varied with stocking density (Figure 8). In groups inoculated with 10^5^ WSSV genome copies/shrimp, donor shrimp exhibited 100% cumulative mortality regardless of stocking density. In recipient shrimp, the first WSSV-associated mortality was observed at 9 dpi in the low-density group and at 5 dpi in the high-density group, and cumulative mortality increased from 35.00% to 86.67% (Figure 8). In groups inoculated with 10^3^ WSSV genome copies/shrimp, cumulative mortality of donor shrimp increased from 58.33% in the low-density group to 79.17% in the high-density group. In recipient shrimp, the first WSSV-associated mortality was observed at 11 dpi in the low-density group and at 6 dpi in the high-density group, and cumulative mortality increased from 20.00% to 60.00% (Figure 8).

#### 3.4.2. WSSV Dynamics in Seawater and Recipient Shrimp During Cohabitation Challenge

**Figure 9:**
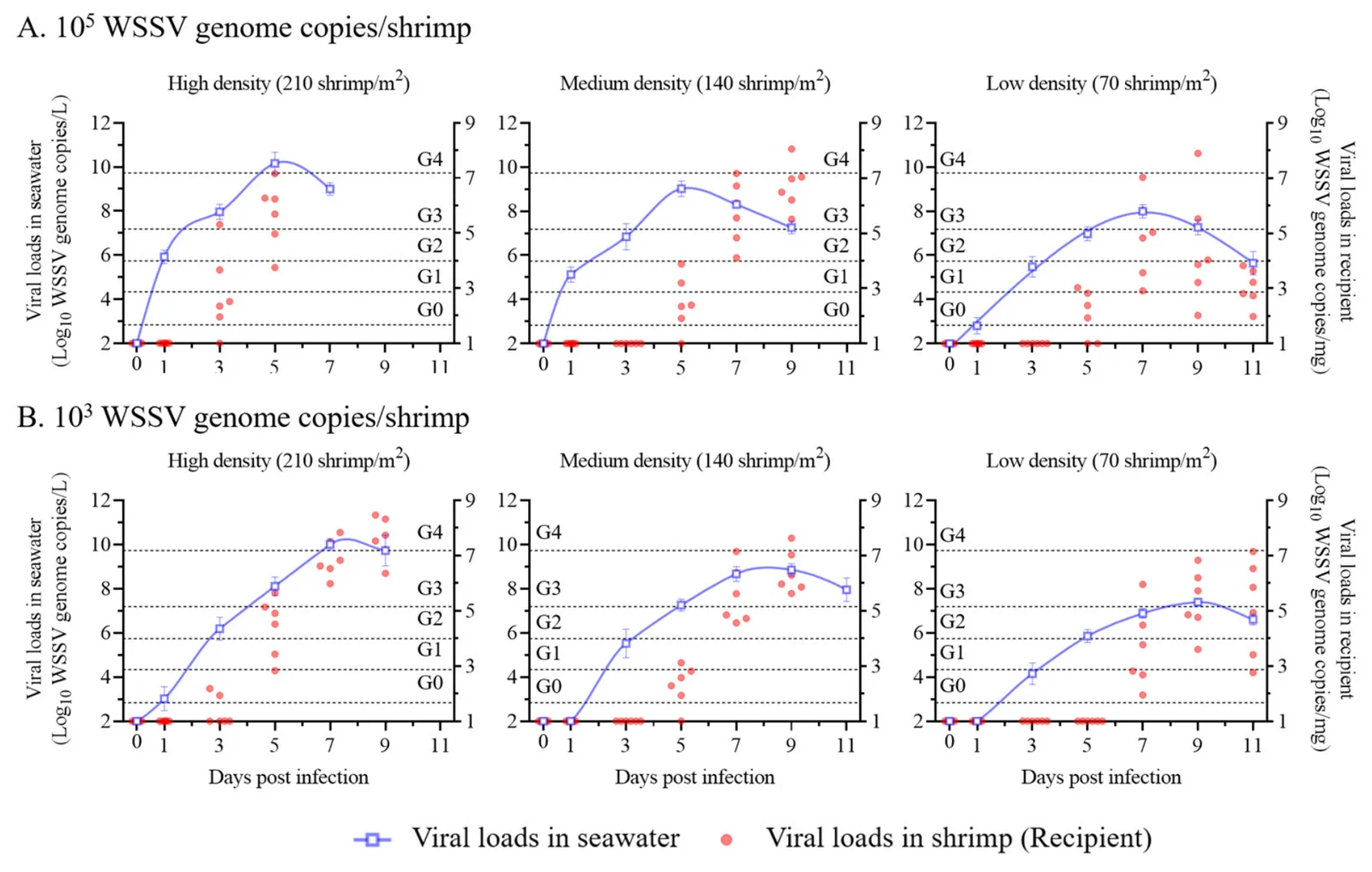
Time-course of viral loads in seawater and recipient shrimp during the cohabitation challenge with WSSV-inoculated donor shrimp (Experiment 4) at 25 °C. Donor shrimp (*n* = 12 per group) were intramuscularly injected with 0.1 mL of diluted WSSV-Te-14 at doses of (**A**) 10^5^ or (**B**) 10^3^ WSSV genome copies/shrimp. Viral loads in seawater and recipient shrimp (*n* = 3 per sampling day) were determined at 1, 3, 5, 7, 9, and 11 days post-infection using duplicate tests. Seawater viral loads were determined from 500-mL rearing seawater samples using the I·D method and are presented as log_10_ WSSV genome copies/L, whereas shrimp viral loads are presented as log_10_ WSSV genome copies/mg. WSSV severity grades were classified according to previously established viral load criteria for whiteleg shrimp [41], and horizontal dashed lines indicate the range of each disease severity grade. I·D, iron flocculation–direct elution; WSSV, white spot syndrome virus.

At 25 °C, in groups inoculated with 10^5^ WSSV genome copies/shrimp, WSSV was detected in seawater at 1 dpi regardless of stocking density. WSSV infection in recipient shrimp was first detected at 5 dpi in the low-density group and at 3 dpi in the high-density group, when seawater viral loads were 10^6.98±0.31^ and 10^7.96±0.34^ WSSV genome copies/L, respectively (Figure 9 and Table 3). In groups inoculated with 10^3^ WSSV genome copies/shrimp, WSSV appeared in seawater earlier in the high-density group than in the low-density group. Recipient-shrimp infection was first detected at 7 dpi in the low-density group and at 3 dpi in the high-density group, when seawater viral loads were 10^6.88±0.22^ and 10^6.19±0.51^ WSSV genome copies/L, respectively (Figure 9 and Table 3).

**Figure 10:**
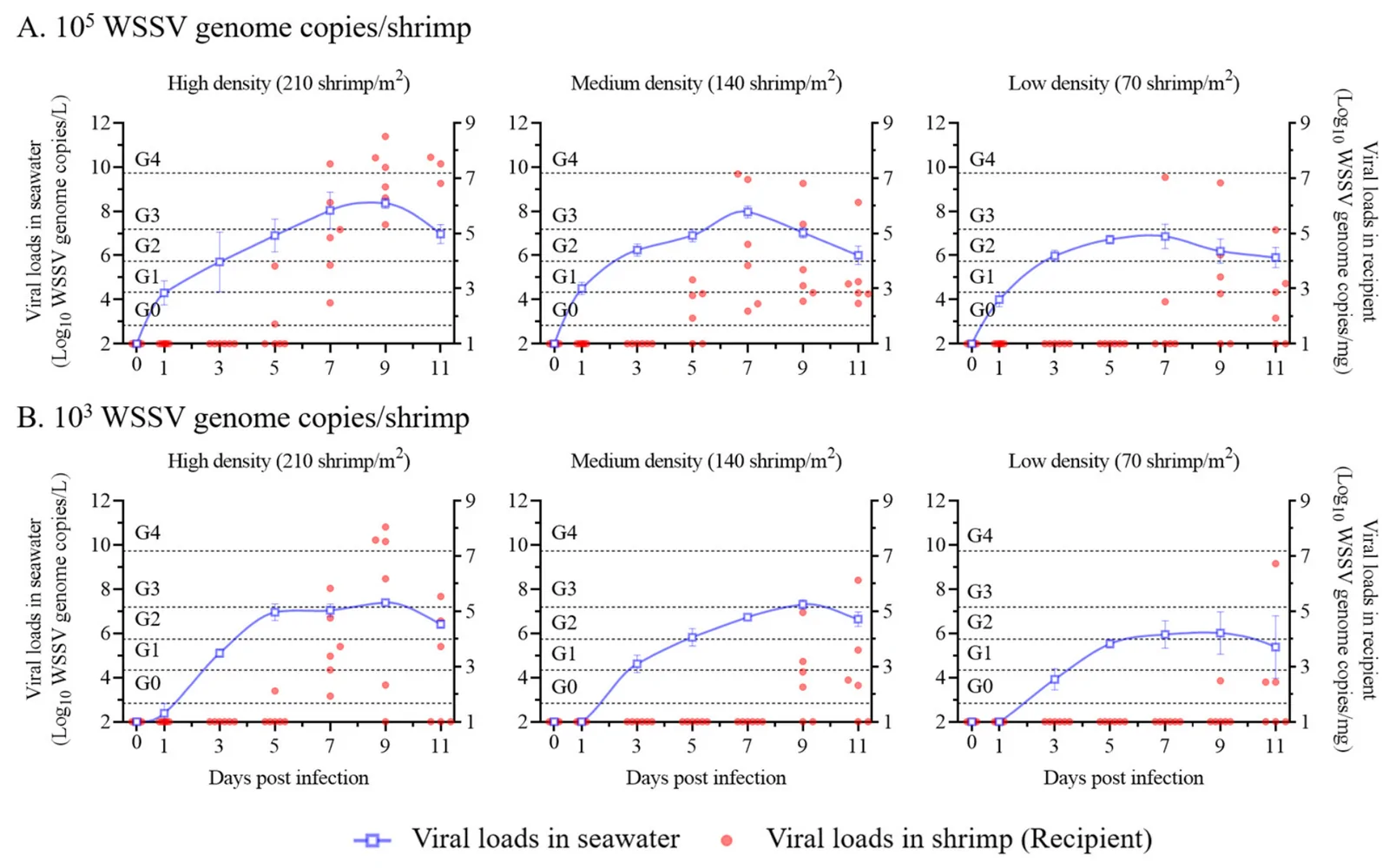
Time-course of viral loads in seawater and recipient shrimp (*n* = 3 per sampling day) during the cohabitation challenge with WSSV-inoculated donor shrimp (Experiment 4) at 30 °C. Donor shrimp (*n* = 12 per group) were intramuscularly injected with 0.1 mL of diluted WSSV-Te-14 at doses of (**A**) 10^5^ or (**B**) 10^3^ WSSV genome copies/shrimp. WSSV severity grades were classified according to previously established viral load criteria for whiteleg shrimp [41], and horizontal dashed lines indicate the range of each disease severity grade. I·D, iron flocculation–direct elution; WSSV, white spot syndrome virus.

At 30 °C, in groups inoculated with 10^5^ WSSV genome copies/shrimp, WSSV was detected in seawater at 1 dpi regardless of stocking density. WSSV infection in recipient shrimp was first detected at 7 dpi in the low-density group and at 5 dpi in the high-density group, when seawater viral loads were 10^9.02±0.35^ and 10^6.90±0.75^ WSSV genome copies/L, respectively (Figure 10 and Table 3). In groups inoculated with 10^3^ WSSV genome copies/shrimp, recipient-shrimp infection was first detected at 9 dpi in the low-density group and at 5 dpi in the high-density group, when seawater viral loads were 10^6.01±0.96^ and 10^6.95±0.38^ WSSV genome copies/L, respectively (Figure 10 and Table 3). Detailed time-course values are shown in Table S5.

#### 3.4.3. Variability in Horizontal Transmission of WSSV During Cohabitation Challenges

**Figure 11:**
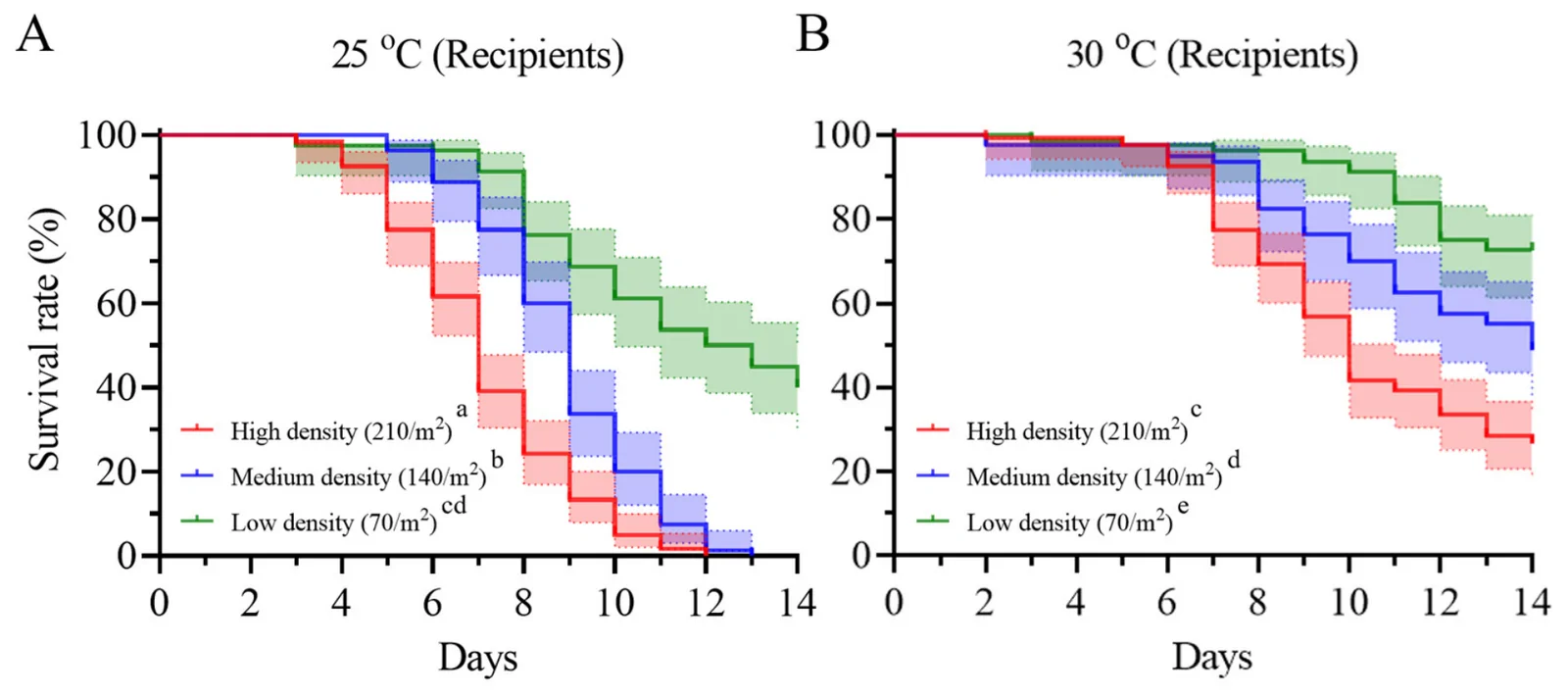
Kaplan–Meier survival curves for recipient shrimp in the cohabitation challenge (Experiment 4) at (**A**) 25 °C and (**B**) 30 °C. The dotted lines indicate the 95% confidence intervals. Survival analysis was performed using pooled data from groups with identical water temperature and stocking density conditions, irrespective of the inoculation dose administered to the donors (10^5^ and 10^3^ WSSV genome copies/shrimp). Statistical significance was determined using the log-rank test, followed by Holm’s pairwise comparison test (*p* < 0.05). Different lowercase letters indicate significant differences among groups, whereas groups sharing the same letter are not significantly different. WSSV, white spot syndrome virus.

Kaplan–Meier survival curves were constructed from recipient shrimp mortality data to evaluate horizontal WSSV transmission under different water temperatures and stocking densities (Figure 11). For this analysis, recipient-shrimp mortality data from the two donor-inoculation doses were pooled within each temperature–density condition. The time to 50% mortality was used as a descriptive indicator of mortality progression in recipient shrimp. At 25 °C, survival probabilities of recipient shrimp were 0.40, 0.00, and 0.00 in the low-, medium-, and high-density groups, respectively; corresponding times to 50% mortality were 12.5, 9, and 7 dpi (Figure 11). At 30 °C, survival probabilities were 0.73, 0.49, and 0.27 in the low-, medium-, and high-density groups, respectively. Times to 50% mortality were 14 and 10 dpi in the medium- and high-density groups, respectively; 50% mortality was not reached in the low-density group within the 14-day observation period (Figure 11). Significant differences in mortality patterns were observed among most groups; however, the 25 °C low-density group did not differ significantly from the 30 °C medium- and high-density groups (Figure 11).

## 4. Discussion

Waterborne transmission is widely regarded as the principal horizontal transmission route of WSSV in penaeid shrimp [28]. Shrimp aquaculture is predominantly practiced in pond-based systems [50]; therefore, seawater exchange may constitute a major biosecurity risk factor for the introduction of pathogens from external sources [11,39]. Under these culture conditions, shrimp are continuously exposed to seasonal environmental fluctuations, particularly variations in water temperature [3]. As shrimp culture systems have become increasingly intensive to improve production yields [51,52], stocking density has emerged as an important husbandry factor influencing disease risk [17,25]. On this basis, we evaluated the susceptibility of whiteleg shrimp to WSSV under different water temperature and stocking density conditions using immersion and cohabitation challenge models. The results demonstrated that WSSV susceptibility, disease progression, and the waterborne MID were affected by water temperature, stocking density, viral concentration, and exposure duration. These findings indicate that waterborne transmission risk should be interpreted in relation to environmental and exposure conditions, rather than viral concentration alone.

Although intensive shrimp culture may improve production, it may also impose chronic physiological stress on shrimp through water quality deterioration and increased competition under crowded conditions [19,20,21,22]. One important consequence is oxidative stress, which results from an imbalance between oxidant production and antioxidant defense [53,54,55,56]. Accordingly, antioxidant-related markers such as *SOD* and *CAT*, together with stress-response proteins including *HSP*s, have been used as indicators of crowding-induced physiological stress [55,56,57]. In Exp. 1, whiteleg shrimp reared at stocking densities of 70, 140, and 210 shrimp/m^2^ for 28 days exhibited density-dependent changes in THC and relative expression levels of *SOD*, *CAT*, and *HSP70* (Figure 2). The high-density group was characterized by reduced expression of antioxidant-related genes (*SOD* and *CAT*), increased *HSP70* expression, and a more pronounced reduction in THC (Figure 2). Several previous studies have reported reduced *SOD* and *CAT* activities and increased *HSP70* expression in whiteleg shrimp at stocking densities exceeding 100 shrimp/m^2^ [54,56]. Some previous studies have reported no clear effect of stocking density on physiological indicators [26,58], possibly because the density ranges examined were relatively low and generally below 100 shrimp/m^2^ [59]. Collectively, these findings suggest that the stocking density conditions used in the present study, particularly 140 and 210 shrimp/m^2^, were associated with changes in physiological stress-related indicators in whiteleg shrimp. Such physiological alterations may help explain the observed differences in WSSV susceptibility.

Several studies have shown that high stocking density may aggravate infection outcomes in aquatic animals. Density-dependent increases in mortality or transmission have been reported for several bacterial and viral pathogens in cultured fish [60,61]. Similar patterns have been reported in whiteleg shrimp challenged with bacterial and viral pathogens, including *Vibrio* spp., infectious myonecrosis virus, and WSSV [22,27,62]. These findings suggest that high stocking density may aggravate infection outcomes, potentially through stress-related physiological alterations and reduced host resistance [22,27,62]. However, more rapid disease progression under high-density conditions may also reflect density-dependent changes in transmission-related processes. In this regard, Apún-Molina et al. [26] reported no significant differences in physiological condition among shrimp reared at different stocking densities in the absence of infection, whereas such differences became evident following WSSV infection. Furthermore, previous studies have reported a density-dependent increase in the probability of WSSV infection, attributed to a higher likelihood of contact among shrimp and subsequent pathogen transmission under intensive culture conditions [24,63]. In the present study, higher stocking-density conditions were associated with increased cumulative mortality and more rapid disease progression regardless of water temperature in Exp. 2 (Figure 3, Figure 4 and Figure 5). At 25 °C, the MID under short-term immersion exposure was 10-fold lower in the high-density group than in the low- and medium-density groups (Table 1). In the cohabitation challenge (Exp. 4), survival probability of recipient shrimp decreased significantly with increasing stocking density at both 25 and 30 °C (Figure 11). WSSV loads in seawater tended to be higher at greater stocking densities, regardless of water temperature, in both Exps. 2 and 4 (Table 1 and Table 3). Taken together, these results suggest that higher stocking-density conditions were associated with increased WSSV susceptibility and accelerated disease progression in whiteleg shrimp, irrespective of water temperature. These responses may reflect combined effects of stress-related physiological alterations and increased opportunities for WSSV transmission among individuals under intensified rearing conditions. Although direct immune endpoints were not examined in the present study, the consistent changes in physiological indicators and infection-related outcomes support the interpretation that intensified rearing conditions may reduce resistance to WSSV and enhance waterborne transmission. Further studies incorporating direct immune-related endpoints are needed to clarify the underlying mechanisms.

Water temperature is one of the principal environmental factors influencing WSSV pathogenicity in cultured shrimp [3,12,13,64]. Previous studies have indicated that the optimal temperature range for WSSV propagation is 23–29 °C, with pathogenicity declining markedly outside this range [14]. This temperature-dependent pattern may partly explain the higher susceptibility observed at 25 °C than at 30 °C in the present study. Because 25 °C falls within the temperature range favorable for WSSV propagation [14,15], viral replication after waterborne exposure may have proceeded more efficiently at this temperature. This may have promoted earlier infection establishment, higher viral loads, and more rapid disease progression. By contrast, 30 °C represents a warmer condition near or slightly above the upper range favorable for WSSV propagation. This condition may therefore have attenuated WSSV pathogenicity or delayed early infection establishment, rather than completely inhibiting infection. For example, reduced expression of the WSSV *VP28* gene has been reported at 30 °C [15], and similar changes in *VP28* expression under high-temperature conditions have been implicated in decreased WSSV pathogenicity [12,13,65]. Although relatively few studies have examined WSSV susceptibility under different water temperature conditions using immersion or cohabitation challenge models, available evidence supports a temperature-dependent pattern in host susceptibility and waterborne transmission [15,66]. This pattern suggests that warmer rearing conditions near or slightly above the upper limit of the optimal WSSV propagation range may reduce the efficiency of WSSV replication or infection establishment during the early phase of infection [12,13,14,15,65]. Therefore, similar temperature-dependent mechanisms may have contributed to the reduced WSSV susceptibility observed at 30 °C in the present study.

The effect of water temperature should not be considered in isolation, however, as WSD transmission risk may remain substantial when other environmental or husbandry factors act concurrently, even outside the optimal temperature range for WSSV replication. Gao et al. [54] reported no significant difference in viral loads between WSSV-infected shrimp reared at 25 and 30 °C under high-salinity conditions (35‰). Another study reported that under intensive culture conditions (210 shrimp/m^2^), mortality in WSSV-infected shrimp reared at 20 or 30 °C did not differ significantly from that in shrimp reared at 70 shrimp/m^2^ under more permissive temperature conditions, such as 25 °C or fluctuating temperatures [24]. At an identical density, shrimp exhibited higher cumulative mortality and more rapid disease progression at 25 °C than at 30 °C in Exp. 2 (Figure 3, Figure 4 and Figure 5). However, when the combined effects of water temperature and stocking density were considered, the MID was identical and mortality patterns of recipient shrimp did not differ significantly between the low-density group at 25 °C and the high-density group at 30 °C (Table 2 and Figure 11). These findings indicate that the reduced WSSV susceptibility observed under warmer conditions should not be interpreted as a temperature effect alone. Although warmer conditions may limit viral replication or delay infection establishment, this effect may be offset by intensified husbandry conditions that increase stress-related physiological changes or transmission opportunities (Figure 11). Therefore, WSD risk assessment under practical farming conditions should consider the combined effects of water temperature and stocking density, rather than interpreting temperature-dependent susceptibility alone.

Determining the waterborne MID may provide important information for disease transmission risk assessment in aquatic animals [38]. However, because no established cell line is available for the propagation and titration of WSSV, the experimental determination of the MID for this virus remains technically challenging [67]. Previous immersion challenge studies have shown that relatively high concentrations, often exceeding 10^7^ WSSV genome copies/L, are generally required to induce waterborne infection under short-term exposure conditions, typically within 24 h [30,31]. Meanwhile, infection may also occur under prolonged or repeated exposure conditions at viral doses that are insufficient to induce infection during short-term exposure [15,68,69]. As discussed above, susceptibility to WSSV may vary according to environmental conditions, particularly water temperature and stocking density. Accordingly, we determined the waterborne MID under different water temperature and stocking density conditions to provide a basis for a more field-relevant assessment of WSSV transmission risk, with particular emphasis on how these factors modify the infective dose and subsequent infection outcome. In Exp. 2, 10^7^ WSSV genome copies/L was insufficient to induce infection in all groups except the high-density group at 25 °C (Figure 3). Under continuous exposure conditions in Exp. 3, WSSV infection was observed at lower challenge doses, although the first detection of infection was delayed as the administered dose decreased (Figure 6). The MID was estimated to be 10^5^ and 10^4^ WSSV genome copies/L at low and high stocking densities, respectively, at 25 °C and 10^6^ and 10^5^ WSSV genome copies/L, respectively, at 30 °C (Table 2). In the cohabitation challenge (Exp. 4), transmission to recipient shrimp was observed in low-density groups at both 25 and 30 °C when donor shrimp were inoculated with 10^3^ WSSV genome copies/shrimp (Table 3). At those time points, WSSV loads in seawater were 10^6.88±0.22^ and 10^6.01±0.96^ WSSV genome copies/L at 25 and 30 °C, respectively, below the MID observed under short-term immersion exposure. Time-course measurements of WSSV loads in seawater indicated that recipient shrimp were continuously exposed from 3 dpi onward (Table 3). Taken together, these findings indicate that the waterborne MID of WSSV should be interpreted not only in relation to viral concentration but also in relation to exposure duration and environmental conditions.

As noted above, the inflow of rearing water from surrounding coastal areas is regarded as one of the principal routes by which pathogens may be introduced into shrimp ponds. Although WSSV may decay relatively rapidly in seawater, its persistence under field conditions may be prolonged through complex interactions with reservoirs and vectors [32,33,34]. Several studies have reported significant associations between WSD occurrence and water-related management practices, including the use of shared water sources [11,39], water exchange practices [25], and water storage reservoirs [17,40]. Methods for concentrating WSSV from environmental waters may provide a basis for quantitative monitoring of WSSV in surrounding coastal waters [33], thereby supporting prevention of pathogen introduction via water exchange and enabling risk assessment. In the present study, immersion and cohabitation challenge models were used to evaluate WSSV susceptibility under different water temperatures and stocking densities. Although these findings should not be interpreted as absolute thresholds for field application, they indicate that prolonged exposure and intensive rearing conditions may increase WSD transmission risk, even at viral concentrations below those generally associated with infection under short-term exposure (Table 2). Accordingly, the risk associated with incoming coastal water should be assessed in relation not only to viral concentration but also to exposure duration and rearing conditions. These results provide an experimental and quantitative basis for assessing WSD transmission risk associated with the introduction of WSSV through coastal water under conditions relevant to pond-based shrimp culture. Furthermore, when combined with monitoring of WSSV in seawater before stocking or prior to water exchange, the quantitative information obtained in this study may serve as a useful reference for evaluating transmission risk and may contribute to reducing the occurrence and spread of WSD in shrimp farms.

Several considerations should be noted when interpreting the results. First, different stocking densities were achieved using tanks with different bottom areas and working volumes. Therefore, potential tank-size-related effects, including differences in available space, water volume, water movement, and WSSV particle distribution, cannot be completely excluded. However, key experimental conditions, including water depth, temperature, feeding, and water-exchange procedures, were standardized across treatments. Thus, the observed responses should be interpreted as outcomes under controlled tank-based density conditions. Second, although duplicate tanks were used in the challenge experiments, viral-load analyses were based on a limited number of shrimp sampled at each time point. This sampling design allowed repeated time-course monitoring across multiple temperature, density, dose, and exposure-duration conditions, but it may not fully capture individual-level variation in WSSV infection dynamics. In addition, direct immune parameters, such as phenoloxidase activity, antimicrobial peptide expression, or hemocyte function, were not examined; therefore, the specific immune mechanisms underlying increased WSSV susceptibility under higher-density conditions should be interpreted cautiously. Nevertheless, the major patterns observed in this study were supported by multiple endpoints, including cumulative mortality, WSSV detection in shrimp, seawater viral loads, MID determination, and cohabitation transmission outcomes. Further studies using identical tank dimensions, larger numbers of replicate tanks or sampled individuals, direct immune-related endpoints, and larger-scale pond-based systems would be useful to validate and refine the quantitative estimates reported here.

## 5. Conclusions

This study demonstrates that water temperature, stocking density, viral concentration, and exposure duration collectively influence the susceptibility of whiteleg shrimp to waterborne WSSV. The waterborne MID of WSSV varied according to water temperature and stocking density, and continuous exposure resulted in transmission even at viral concentrations that were insufficient to induce infection under short-term exposure conditions. These findings indicate that the WSD transmission risk associated with incoming coastal water should not be evaluated solely on the basis of viral concentration, but should also consider exposure duration and rearing conditions. Overall, this study provides a quantitative experimental basis for assessing WSSV transmission risk under conditions relevant to pond-based shrimp culture and may support risk-based management of incoming coastal water when combined with seawater WSSV monitoring.

## Figures and Tables

**Table 1:** Summary of WSSV detection and viral-load dynamics in shrimp and seawater after immersion challenge in Experiment 2 (short-term immersion exposure).

Immersion Dose ^1^	Temp. (°C)	Stocking Density	Seawater Viral Load at First WSSV Detection in Shrimp ^2^	Shrimp		
				First WSSV Detection (dpi ^3^)	Peak Viral Load ^4^	
					Time (dpi)	Median [IQR] (Severity Grade)
10^8^	25 °C	High density (210/m^2^)	10^6.94±0.28^	3	9	10^7.52^ [10^7.17^–10^7.87^] (G3–G4)
		Medium density (140/m^2^)	10^6.77±0.58^	3	9	10^6.57^ [10^6.06^–10^6.98^] (G3)
		Low density (70/m^2^)	10^6.37±0.08^	3	9	10^4.78^ [10^4.17^–10^5.23^] (G2–G3)
	30 °C	High density (210/m^2^)	10^6.21±0.28^	3	9	10^4.26^ [10^3.72^–10^4.81^] (G1–G2)
		Medium density (140/m^2^)	10^6.32±0.47^	3	9	10^5.36^ [10^3.51^–10^7.22^] (G1–G4)
		Low density (70/m^2^)	10^4.84±0.61^	5	7	10^3.66^ [10^3.51^–10^3.81^] (G1)
10^7^	25 °C	High density (210/m^2^)	10^6.24±0.09^	3	7	10^7.40^ [10^7.18^–10^7.88^] (G4)
	Others			Not confirmed ^5^		

^1^ WSSV genome copies/L. ^2^ Viral loads in seawater are presented as mean ± standard deviation (SD) WSSV genome copies/L. ^3^ dpi, Days post-infection. ^4^ Viral loads in shrimp are presented as median (interquartile range; IQR) WSSV genome copies/mg. ^5^ Among groups exposed to 10^7^ WSSV genome copies/L, WSSV infection was confirmed only in the high-density group at 25 °C.

**Table 2:** Summary of cumulative mortality and time-course WSSV detection in shrimp in Experiment 3 (continuous immersion exposure).

Stock Density	Immersion Dose ^1^	Temperature (°C)					
		25 °C			30 °C		
		First WSSV Detection in Shrimp (dpi ^2^)	Cumulative Mortality	Infection Rate (%) (Inf./Tot. ^3^)	First WSSV Detection in Shrimp (dpi)	Cumulative Mortality	Infection Rate (%) (Inf./Tot.)
High density (210/m^2^)	10^6^	3	83.33 ± 10.10	100.00 ± 0.00 (42/42)	3	100.00 ± 0.00	100.00 ± 0.00 (42/42)
	10^5^	5	59.52 ± 10.10	76.19 ± 13.47 (32/42)	5	69.05 ± 16.84	92.86 ± 10.10 (39/42)
	10^4^	7	40.48 ± 3.37	54.76 ± 3.37 (23/42)	7	40.48 ± 3.37	61.90 ± 6.73 (26/42)
	10^3^	U.D. ^4^	7.14 ± 3.37	0.00 ± 0.00 (0/42)	U.D.	0.00 ± 0.00	0.00 ± 0.00 (0/42)
	10^2^	U.D.	0.00 ± 0.00	0.00 ± 0.00 (0/42)	U.D.	4.76 ± 0.00	0.00 ± 0.00 (0/42)
	Control	U.D.	7.14 ± 3.37	0.00 ± 0.00 (0/42)	U.D.	4.76 ± 6.73	0.00 ± 0.00 (0/42)
Low density (70/m^2^)	10^7^	3	100.00 ± 0.00	100.00 ± 0.00 (28/28)	3	67.86 ± 5.05	100.00 ± 0.00 (28/28)
	10^6^	5	89.29 ± 15.15	89.29 ± 15.15 (25/28)	7	32.14 ± 5.05	50.00 ± 0.00 (14/28)
	10^5^	7	50.00 ± 20.20	50.00 ± 20.20 (14/28)	U.D.	0.00 ± 0.00	0.00 ± 0.00 (0/28)
	10^4^	U.D.	0.00 ± 0.00	0.00 ± 0.00 (0/28)	U.D.	3.57 ± 5.05	0.00 ± 0.00 (0/28)
	10^3^	U.D.	0.00 ± 0.00	0.00 ± 0.00 (0/28)	U.D.	0.00 ± 0.00	0.00 ± 0.00 (0/28)
	Control	U.D.	0.00 ± 0.00	0.00 ± 0.00 (0/28)	U.D.	3.57 ± 5.05	0.00 ± 0.00 (0/28)

^1^ WSSV genome copies/L. ^2^ dpi, Days post-infection. ^3^ WSSV infection was confirmed using real-time PCR according to the protocol described by Zhou and Lightner. Samples with WSSV genome copy numbers equal to or greater than the 95% limit of detection described in Section 2.3 were considered WSSV-positive. ^4^ U.D., undetermined.

**Table 3:** Summary of WSSV detection and viral-load dynamics in seawater and recipient shrimp during the cohabitation challenge in Experiment 4.

Injection Dose (Donor) ^1^	Temp. (°C)	Stocking Density	Seawater		First WSSV Detection in Recipient Shrimp (dpi)
			WSSV Detection Period (dpi ^2^)	Viral Load at First WSSV Detection in Recipient Shrimp ^3^	
10^5^	25 °C	High density (210/m^2^)	1–7 ^4^	10^7.96±0.34^	3
		Medium density (140/m^2^)	1–9 ^4^	10^9.02±0.35^	5
		Low density (70/m^2^)	1–11	10^6.98±0.31^	5
	30 °C	High density (210/m^2^)	1–11	10^6.90±0.75^	5
		Medium density (140/m^2^)	1–11	10^6.91±0.29^	5
		Low density (70/m^2^)	1–11	10^6.86±0.55^	7
10^3^	25 °C	High density (210/m^2^)	1–9 ^4^	10^6.19±0.51^	3
		Medium density (140/m^2^)	3–9 ^4^	10^7.25±0.27^	5
		Low density (70/m^2^)	3–11	10^6.88±0.22^	7
	30 °C	High density (210/m^2^)	1–11	10^6.95±0.38^	5
		Medium density (140/m^2^)	3–11	10^7.31±0.22^	9
		Low density (70/m^2^)	3–11	10^6.01±0.96^	9

^1^ WSSV genome copies/shrimp. ^2^ dpi, Days post-infection. ^3^ Viral loads in seawater are presented as mean ± standard deviation (SD) WSSV genome copies/L. ^4^ For groups in which all shrimp died before 11 dpi, seawater sampling was discontinued at the time of group termination.

## Data Availability

The original contributions presented in this study are included in the article and Supplementary Materials. Further inquiries can be directed to the corresponding author.

## Supplementary Materials

The following supporting information can be downloaded at: https://www.mdpi.com/article/10.3390/ani16182855/s1, Figure S1: Diagnostic plots of the two-way ANOVA models used in Experiment 1; Table S1: Primer and probe used in this study [30,70,71,72]; Table S2: Correlation between WSSV severity grade and quantitative analysis [41]; Table S3: Detailed time-course dynamics of WSSV in seawater and shrimp, and disease severity grades in Experiment 2 (short-term immersion exposure); Table S4. Cumulative mortality in challenged shrimp and detailed WSSV infection in shrimp sampled at different time points in Experiment 3 (continuous immersion exposure); Table S5. Detailed time-course dynamics of WSSV in seawater and shrimp, and disease severity grades in Experiment 4 (cohabitation challenge).

## Abbreviations

The following abbreviations are used in this manuscript:

WSSV	White spot syndrome virus
WSD	White spot disease
*MID*	Minimum infective dose
*SOD*	Superoxide dismutase
*CAT*	Catalase
*HSP70*	Heat shock protein 70
ANOVA	Analysis of variance
PBS	Phosphate-buffered saline
UV	Ultraviolet
I·D	Iron flocculation–direct elution
