# Supplementary material for: Effects of Water Temperature, Stocking Density, and Exposure Period on the Susceptibility of Whiteleg Shrimp (Penaeus vannamei) to Waterborne White Spot Syndrome Virus
Source: Animals (Basel). 2026 Sep 10;16(18):2855. doi: 10.3390/ani16182855 (PMC13603552; doi:10.3390/ani16182855)
Supplement: Supplementary file 1 [file animals-16-02855-s001.zip › animals-4537293-supplementary.pdf]

Table S1. Primer and probe used in this study.

Target gene	Primer	Sequence (5'-3')	Purpose	Reference
WSSV (*VP* 664)	WSSV 1011F	TGG TCC CGT CCT CAT CTC AG	Quantitative analysis	Durand and Lightner. [30]
	WSSV 1079R	GCT GCC TTG CCG GAA ATT A		
	TaqMan probe	AGC CAT GAA GAA TGC CGT CTA TCA CAC A		
Heat shock protein 70 (*HSP70*)	HSP70-F	CCT CCA GGA CTT CTT CAA CG	Relative gene expression	Zhou et al. [70]
	HSP70-R	GGT CAC GTC CAA CAG CAA C		
Superoxidase dismutase (*SOD*)	SOD-F	ATC CAC CAC ACA AAG CAT CA		Wang et al. [71]
	SOD-R	AGC TCT CGT CAA TGG CTT GT		
Catalase (*CAT*)	CAT-F	ACT CCC ATT GCT GTT CGT		Xu et al. [72]
	CAT-R	ATC CCA ATT TCC TTC TTC TG		
Beta actin (*β-actin*)	β-actin-F	CGA GGT ATC CTC ACC CTG A		
	β-actin-R	CGG AGC TCG TTG TAG AAG G		

Table S2. Correlation between WSSV severity grade and quantitative analysis

WSSV severity grade	WSSV genome copies/mg	C_T value	Reference
Negative	Below than $10^{1.67}$	Greater than 36.43	Kim et al. [41]
G0	$10^{1.67}$–$10^{2.87}$	36.43–32.13	
G1	$10^{2.87}$–$10^{3.99}$	32.13–28.09	
G2	$10^{3.99}$–$10^{5.15}$	28.09–23.97	
G3	$10^{5.15}$–$10^{7.18}$	23.97–16.70	
G4	Greater than $10^{7.18}$	Below than 16.70	

Table S3. Detailed time-course dynamics of WSSV in seawater and shrimp, and disease severity grades in Experiment 2 (short-term immersion exposure)

Temperature	Immersion doses [1]	Stocking density	WSSV dynamics	Days					
				1	3	5	7	9	11
25°C	10^{8}	High density (210/m²)	Viral loads in seawater [2]	$10^{7.61±0.23}$	$10^{6.94±0.28}$	$10^{8.49±0.38}$	$10^{8.87±0.24}$	$10^{7.99±0.27}$	$10^{6.60±0.37}$
			Viral loads in shrimp [3] (Inf./Tot.)	U.D. [5] (0/6)	$10^{4.78}$ [$10^{4.01}$–$10^{5.98}$] (6/6)	$10^{5.51}$ [$10^{5.01}$–$10^{6.06}$] (6/6)	$10^{7.42}$ [$10^{7.18}$–$10^{8.01}$] (6/6)	$10^{7.52}$ [$10^{7.17}$–$10^{7.87}$] (2/6)	N.D. [6]
			Severity grades of shrimp [4]	–	G2–G3	G2–G3	G4	G3–G4	–
		Medium density (140/m²)	Viral loads in seawater	$10^{7.57±0.27}$	$10^{6.77±0.58}$	$10^{7.84±0.33}$	$10^{7.52±0.26}$	$10^{6.63±0.27}$	$10^{4.86±0.53}$
			Viral loads in shrimp (Inf./Tot)	U.D. (0/6)	$10^{2.54}$ [$10^{2.17}$–$10^{2.92}$] (4/6)	$10^{4.94}$ [$10^{3.45}$–$10^{6.49}$] (6/6)	$10^{5.36}$ [$10^{4.16}$–$10^{6.36}$] (6/6)	$10^{6.57}$ [$10^{6.06}$–$10^{6.98}$] (6/6)	N.D.
			Severity grades of shrimp	–	G0–G1	G1–G3	G2–G3	G3	–
		Low density (70/m²)	Viral loads in seawater	$10^{7.56±0.21}$	$10^{6.37±0.08}$	$10^{6.34±0.44}$	$10^{7.41±0.27}$	$10^{6.71±0.19}$	$10^{5.91±0.29}$
			Viral loads in shrimp (Inf./Tot)	U.D. (0/6)	$10^{2.72}$ (1/6)	$10^{3.72}$ [$10^{3.58}$–$10^{4.31}$] (3/6)	$10^{4.58}$ [$10^{3.97}$–$10^{5.43}$] (5/6)	$10^{4.78}$ [$10^{4.17}$–$10^{5.23}$] (6/6)	$10^{3.83}$ [$10^{3.10}$–$10^{4.68}$] (6/6)
			Severity grades of shrimp	–	G0	G1–G2	G1–G3	G2–G3	G1–G2

[1] WSSV genome copies/L.; [2] Viral loads in seawater is presented as mean ± standard deviation (SD) WSSV genome copies/L.; [3] Viral loads in shrimp is presented as median (interquartile range; IQR) WSSV genome copies/mg.; [4] WSSV severity grades were determined based on the WSSV genome copies in shrimp (Kim et al., 2023).; [5] U.D., undetermined.; [6] N.D., Not done.

Table S3. (*Continued*)

Temperature	Immersion dose [1]	Stocking density	WSSV dynamics	Days					
				1	3	5	7	9	11
25°C	10^7	High density (210/m^2)	Viral loads in seawater [2]	$10^{6.47±0.27}$	$10^{6.24±0.09}$	$10^{6.86±0.25}$	$10^{9.16±0.25}$	$10^{7.71±0.17}$	$10^{6.14±0.30}$
			Viral loads in shrimp [3] (Inf./Tot.)	U.D. [5] (0/6)	$10^{5.03}$ [$10^{4.20}$–$10^{5.48}$] (6/6)	$10^{6.45}$ [$10^{6.33}$–$10^{6.78}$] (6/6)	$10^{7.40}$ [$10^{7.18}$–$10^{7.88}$] (6/6)	$10^{5.04}$ [$10^{3.27}$–$10^{6.81}$] (4/6)	N.D. [6]
			Severity grades of shrimp [4]	–	G2−G3	G3	G4	G1−G3	–
		Medium density (140/m^2)	Viral loads in seawater	$10^{6.87±0.07}$	$10^{5.78±0.30}$	$10^{4.22±0.35}$	$10^{2.92±0.32}$	$10^{2.41±0.08}$	U.D.
			Viral loads in shrimp (Inf./Tot)	U.D. (0/6)	U.D. (0/6)	U.D. (0/6)	U.D. (0/6)	U.D. (0/6)	U.D. (0/6)
			Severity grades of shrimp	–	–	–	–	–	–
		Low density (70/m^2)	Viral loads in seawater	$10^{6.70±0.26}$	$10^{5.72±0.20}$	$10^{3.95±0.37}$	$10^{2.94±0.30}$	$10^{2.38}$	U.D.
			Viral loads in shrimp (Inf./Tot)	U.D. (0/6)	U.D. (0/6)	U.D. (0/6)	U.D. (0/6)	U.D. (0/6)	U.D. (0/6)
			Severity grades of shrimp	–	–	–	–	–	–

[1] WSSV genome copies/L.; [2] Viral loads in seawater is presented as mean ± standard deviation (SD) WSSV genome copies/L.; [3] Viral loads in shrimp is presented as median (interquartile range; IQR) WSSV genome copies/mg.; [4] WSSV severity grades were determined based on the WSSV genome copies in shrimp (Kim et al., 2023).; [5] U.D., undetermined.; [6] N.D., Not done.

Table S3. (*Continued*)

Temperature	Immersion dose [1]	Stocking density	WSSV dynamics	Days					
				1	3	5	7	9	11
30°C	10^8	High density ($210/m^2$)	Viral loads in seawater [2]	$10^{7.66±0.18}$	$10^{6.21±0.28}$	$10^{5.45±0.24}$	$10^{4.99±0.42}$	$10^{4.33±0.27}$	$10^{3.23±0.27}$
			Viral loads in shrimp [3] (Inf./Tot.)	U.D. [5] (0/6)	$10^{3.28}$ [$10^{3.02}$–$10^{3.55}$] (2/6)	$10^{3.12}$ [$10^{2.82}$–$10^{5.89}$] (5/6)	$10^{3.92}$ [$10^{3.10}$–$10^{4.14}$] (5/6)	$10^{4.26}$ [$10^{3.72}$–$10^{4.81}$] (4/6)	$10^{2.56}$ [$10^{2.31}$–$10^{2.81}$] (4/6)
			Severity grades of shrimp [4]	–	G1	G0−G3	G1−G2	G1−G2	G0
		Medium density ($140/m^2$)	Viral loads in seawater	$10^{7.66±0.17}$	$10^{6.32±0.47}$	$10^{6.56±0.24}$	$10^{5.81±0.46}$	$10^{5.17±0.26}$	$10^{3.54±0.16}$
			Viral loads in shrimp (Inf./Tot)	U.D. (0/6)	$10^{2.90}$ (1/6)	$10^{3.21}$ [$10^{3.11}$–$10^{3.95}$] (3/6)	$10^{3.17}$ [$10^{2.86}$–$10^{3.56}$] (3/6)	$10^{5.36}$ [$10^{3.51}$–$10^{7.22}$] (4/6)	$10^{2.72}$ [$10^{2.44}$–$10^{3.06}$] (4/6)
			Severity grades of shrimp	–	G1	G1	G0−G1	G1−G4	G0−G1
		Low density ($70/m^2$)	Viral loads in seawater	$10^{7.74±0.22}$	$10^{5.94±0.38}$	$10^{4.84±0.61}$	$10^{3.61±0.27}$	U.D.	U.D.
			Viral loads in shrimp (Inf./Tot)	U.D. (0/6)	U.D. (0/6)	$10^{3.01}$ [$10^{2.66}$–$10^{3.36}$] (2/6)	$10^{3.66}$ [$10^{3.51}$–$10^{3.81}$] (4/6)	$10^{2.80}$ [$10^{2.28}$–$10^{3.31}$] (3/6)	$10^{2.31}$ [$10^{1.81}$–$10^{2.81}$] (3/6)
			Severity grades of shrimp	–	–	G0−G1	G1	G0−G1	G0

[1] WSSV genome copies/L.; [2] Viral loads in seawater is presented as mean ± standard deviation (SD) WSSV genome copies/L.; [3] Viral loads in shrimp is presented as median (interquartile range; IQR) WSSV genome copies/mg.; [4] WSSV severity grades were determined based on the WSSV genome copies in shrimp (Kim et al., 2023).; [5] U.D., undetermined.; [6] N.D., Not done.

Table S3. (*Continued*)

Temperature	Immersion dose [1]	Stocking density	WSSV dynamics	Days					
				1	3	5	7	9	11
30ºC	10^7	High density (210/m^2)	Viral loads in seawater [2]	$10^{6.46±0.26}$	$10^{5.00±0.39}$	$10^{3.76±0.27}$	$10^{2.71±0.39}$	U.D.[5]	U.D.
			Viral loads in shrimp [3] (Inf./Tot.)	U.D. [5] (0/6)	U.D. (0/6)	U.D. (0/6)	U.D. (0/6)	U.D. (0/6)	U.D. (0/6)
			Severity grades of shrimp [4]	–	–	–	–	–	–
		Medium density (140/m^2)	Viral loads in seawater	$10^{6.59±0.14}$	$10^{5.05±0.43}$	$10^{3.06±0.48}$	$10^{2.39±0.15}$	U.D.	U.D.
			Viral loads in shrimp (Inf./Tot)	U.D. (0/6)	U.D. (0/6)	U.D. (0/6)	U.D. (0/6)	U.D. (0/6)	U.D. (0/6)
			Severity grades of shrimp	–	–	–	–	–	–
		Low density (70/m^2)	Viral loads in seawater	$10^{6.66±0.21}$	$10^{5.49±0.30}$	$10^{3.74±0.36}$	$10^{2.73±0.43}$	U.D.	U.D.
			Viral loads in shrimp (Inf./Tot)	U.D. (0/6)	U.D. (0/6)	U.D. (0/6)	U.D. (0/6)	U.D. (0/6)	U.D. (0/6)
			Severity grades of shrimp	–	–	–	–	–	–

[1] WSSV genome copies/L.; [2] Viral loads in seawater is presented as mean ± standard deviation (SD) WSSV genome copies/L.; [3] Viral loads in shrimp is presented as median (interquartile range; IQR) WSSV genome copies/mg.; [4] WSSV severity grades were determined based on the WSSV genome copies in shrimp (Kim et al., 2023).; [5] U.D., undetermined.; [6] N.D., Not done.

Table S4. Cumulative mortality in challenged shrimp and detailed WSSV infection in shrimp sampled at different time points in Experiment 3 (continuous immersion exposure)

Temperature	Stock density	WSSV genome copies/L	Days post infection (Inf./Tot.) [1]						Cumulative mortality	Infection rate (%) (Inf./Tot.) [2]
			1	3	5	7	9	11		
25 ºC	High density (210 shrimp/m^2)	10^6	0/6	**3/6**	**6/6**	**6/6**	**6/6**	**6/6**	83.33 ± 10.10	100.00 ± 0.00 (42/42)
		10^5	0/6	0/6	0/6	**2/6**	**5/6**	**6/6**	59.52 ± 10.10	76.19 ± 13.47 (32/42)
		10^4	0/6	0/6	0/6	0/6	**4/6**	**4/6**	40.48 ± 3.37	54.76 ± 3.37 (23/42)
		10^3	0/6	0/6	0/6	0/6	0/6	0/6	7.14 ± 3.37	0.00 ± 0.00 (0/42)
		10^2	0/6	0/6	0/6	0/6	0/6	0/6	0.00 ± 0.00	0.00 ± 0.00 (0/42)
		Control	0/6	0/6	0/6	0/6	0/6	0/6	7.14 ± 3.37	0.00 ± 0.00 (0/42)
	Low (70 shrimp/m^2)	10^7	0/6	**6/6**	**6/6**	**6/6**	N.D. [2]	N.D.	100.00 ± 0.00	100.00 ± 0.00 (28/28)
		10^6	0/6	0/6	**2/6**	**2/6**	**6/6**	**6/6**	89.29 ± 15.15	89.29 ± 15.15 (25/28)
		10^5	0/6	0/6	0/6	**2/6**	**2/6**	**4/6**	50.00 ± 20.20	50.00 ± 20.20 (14/28)
		10^4	0/6	0/6	0/6	0/6	0/6	0/6	0.00 ± 0.00	0.00 ± 0.00 (0/28)
		10^3	0/6	0/6	0/6	0/6	0/6	0/6	0.00 ± 0.00	0.00 ± 0.00 (0/28)
		Control	0/6	0/6	0/6	0/6	0/6	0/6	0.00 ± 0.00	0.00 ± 0.00 (0/28)
30 ºC	High (210 shrimp/m^2)	10^7	0/6	**6/6**	**6/6**	**6/6**	N.D.	N.D.	100.00 ± 0.00	100.00 ± 0.00 (42/42)
		10^6	0/6	0/6	**4/6**	**6/6**	**6/6**	N.D.	69.05 ± 16.84	92.86 ± 10.10 (39/42)
		10^5	0/6	0/6	0/6	**1/6**	**6/6**	**5/6**	40.48 ± 3.37	61.90 ± 6.73 (26/42)
		10^4	0/6	0/6	0/6	0/6	0/6	0/6	0.00 ± 0.00	0.00 ± 0.00 (0/42)
		10^3	0/6	0/6	0/6	0/6	0/6	0/6	4.76 ± 0.00	0.00 ± 0.00 (0/42)
		Control	0/6	0/6	0/6	0/6	0/6	0/6	4.76 ± 6.73	0.00 ± 0.00 (0/42)
	Low (70 shrimp/m^2)	10^7	0/6	**3/6**	**3/6**	**6/6**	**5/3**	**6/6**	67.86 ± 5.05	100.00 ± 0.00 (28/28)
		10^6	0/6	0/6	0/6	**2/6**	**4/6**	**4/6**	32.14 ± 5.05	50.00 ± 0.00 (14/28)
		10^5	0/6	0/6	0/6	0/6	0/6	0/6	0.00 ± 0.00	0.00 ± 0.00 (0/28)
		10^4	0/6	0/6	0/6	0/6	0/6	0/6	3.57 ± 5.05	0.00 ± 0.00 (0/28)
		10^3	0/6	0/6	0/6	0/6	0/6	0/6	0.00 ± 0.00	0.00 ± 0.00 (0/28)
		Control	0/6	0/6	0/6	0/6	0/6	0/6	3.57 ± 5.05	0.00 ± 0.00 (0/28)

[1] WSSV infection was confirmed using real-time PCR according to the protocol described by Durand and Lightner. Samples with WSSV genome copy numbers equal to or greater than the 95% limit of detection described in Section 2.3 were considered WSSV-positive.; [2] N.D., Not done.

Table S5. Detailed time-course dynamics of WSSV in seawater and shrimp, and disease severity grades in Experiment 4 (cohabitation challenge)

Temperature	Injection dose (Donor) [1]	Stocking density	WSSV dynamics	Days (Recipient)					
				1	3	5	7	9	11
25°C	10^{5}	High density (210/m^2)	Viral loads in seawater [2]	$10^{5.92±0.30}$	$10^{7.96±0.34}$	$10^{10.16±0.51}$	$10^{9.00±0.29}$	N.D.	N.D.
			Viral loads in shrimp [3] (Inf./Tot.)	U.D. [5] (0/6)	$10^{2.51}$ [$10^{2.35}$–$10^{3.66}$] (5/6)	$10^{5.97}$ [$10^{5.15}$–$10^{6.26}$] (6/6)	N.D. [6]	N.D.	N.D.
			Severity grades of shrimp [4]	–	G0−G3	G1−G4	–	–	–
		Medium density (140/m^2)	Viral loads in seawater	$10^{5.13±0.34}$	$10^{6.85±0.59}$	$10^{9.02±0.35}$	$10^{8.31±0.15}$	$10^{7.26±0.29}$	N.D.
			Viral loads in shrimp (Inf./Tot)	U.D. (0/6)	U.D. (0/6)	$10^{2.40}$ [$10^{2.35}$–$10^{3.20}$] (5/6)	$10^{5.83}$ [$10^{5.02}$–$10^{6.57}$] (6/6)	$10^{6.73}$ [$10^{6.28}$–$10^{7.03}$] (6/6)	N.D.
			Severity grades of shrimp	–	–	G0−G1	G2−G3	G3−G4	–
		Low density (70/m^2)	Viral loads in seawater	$10^{2.81±0.37}$	$10^{5.48±0.46}$	$10^{6.98±0.31}$	$10^{7.99±0.31}$	$10^{7.27±0.34}$	$10^{5.64±0.54}$
			Viral loads in shrimp (Inf./Tot)	U.D. (0/6)	U.D. (0/6)	$10^{2.61}$ [$10^{2.27}$–$10^{2.88}$] (4/6)	$10^{4.93}$ [$10^{3.88}$–$10^{5.63}$] (6/6)	$10^{3.95}$ [$10^{3.38}$–$10^{5.15}$] (6/6)	$10^{3.02}$ [$10^{2.76}$–$10^{3.53}$] (6/6)
			Severity grades of shrimp	–	–	G0−G1	G1−G3	G0−G4	G0−G1

[1] WSSV genome copies/shrimp.; [2] Viral loads in seawater is presented as mean ± standard deviation (SD) WSSV genome copies/L.; [3] Viral loads in shrimp is presented as median (interquartile range; IQR) WSSV genome copies/mg.; [4] WSSV severity grades were determined based on the WSSV genome copies in shrimp (Kim et al., 2023).; [5] U.D., undetermined.; [6] N.D., Not done.

Table S5. (*Continued*)

Temperature	Injection dose (Donor) [1]	Stocking density	WSSV dynamics	Days (Recipient) 1	3	5	7	9	11
25°C	10^3	High density (210/m^2)	Viral loads in seawater [2]	$10^{3.02\pm0.54}$	$10^{6.19\pm0.51}$	$10^{8.12\pm0.42}$	$10^{10.00\pm0.26}$	$10^{9.73\pm0.69}$	N.D. [6]
			Viral loads in shrimp [3] (Inf./Tot.)	U.D. [5] (0/6)	$10^{2.06}$ [$10^{2.00}$–$10^{2.12}$] (2/6)	$10^{4.71}$ [$10^{3.70}$–$10^{5.08}$] (6/6)	$10^{6.73}$ [$10^{6.56}$–$10^{7.33}$] (6/6)	$10^{7.63}$ [$10^{7.29}$–$10^{8.18}$] (6/6)	N.D.
			Severity grades of shrimp [4]	–	G0	G0−G3	G3−G4	G3−G4	–
		Medium density (140/m^2)	Viral loads in seawater	U.D.	$10^{5.52\pm0.64}$	$10^{7.25\pm0.27}$	$10^{8.67\pm0.33}$	$10^{8.86\pm0.27}$	N.D.
			Viral loads in shrimp (Inf./Tot)	U.D. (0/6)	U.D. (0/6)	$10^{2.58}$ [$10^{2.28}$–$10^{2.81}$] (5/6)	$10^{5.23}$ [$10^{4.76}$–$10^{6.19}$] (6/6)	$10^{6.13}$ [$10^{5.89}$–$10^{6.84}$] (6/6)	N.D.
			Severity grades of shrimp	–	–	G0−G1	G2−G3	G3−G4	–
		Low density (70/m^2)	Viral loads in seawater	U.D.	$10^{4.15\pm0.48}$	$10^{5.85\pm0.30}$	$10^{6.88\pm0.22}$	$10^{7.39\pm0.16}$	$10^{6.60\pm0.24}$
			Viral loads in shrimp (Inf./Tot)	U.D. (0/6)	U.D. (0/6)	U.D. (0/6)	$10^{3.29}$ [$10^{2.72}$–$10^{4.30}$] (6/6)	$10^{5.29}$ [$10^{4.79}$–$10^{6.08}$] (6/6)	$10^{5.39}$ [$10^{3.79}$–$10^{6.36}$] (6/6)
			Severity grades of shrimp	–	–	–	G0−G3	G1−G3	G0−G3

[1] WSSV genome copies/shrimp.; [2] Viral loads in seawater is presented as mean ± standard deviation (SD) WSSV genome copies/L.; [3] Viral loads in shrimp is presented as median (interquartile range; IQR) WSSV genome copies/mg.; [4] WSSV severity grades were determined based on the WSSV genome copies in shrimp (Kim et al., 2023).; [5] U.D., undetermined.; [6] N.D., Not done.

Table S5. (*Continued*)

Temperature	Injection dose (Donor) [1]	Stocking density	WSSV dynamics	Days (Recipient)					
				1	3	5	7	9	11
30°C	10^5	High density ($210/m^2$)	Viral loads in seawater [2]	$10^{4.30\pm0.55}$	$10^{5.70\pm1.35}$	$10^{6.90\pm0.75}$	$10^{8.04\pm0.83}$	$10^{8.36\pm0.22}$	$10^{6.97\pm0.43}$
			Viral loads in shrimp [3] (Inf./Tot.)	U.D. [5]<br>(0/6)	U.D.<br>(0/6)	$10^{2.77}$<br>[$10^{2.24}$–$10^{3.29}$]<br>(2/6)	$10^{4.99}$<br>[$10^{4.10}$–$10^{5.87}$]<br>(4/6)	$10^{7.04}$<br>[$10^{6.38}$–$10^{7.65}$]<br>(6/6)	$10^{7.51}$<br>[$10^{7.16}$–$10^{7.63}$]<br>(6/6)
			Severity grades of shrimp [4]	–	–	G0−G1	G0−G4	G3−G4	G3−G4
		Medium density ($140/m^2$)	Viral loads in seawater	$10^{4.50\pm0.27}$	$10^{6.24\pm0.28}$	$10^{6.91\pm0.29}$	$10^{7.97\pm0.27}$	$10^{7.03\pm0.22}$	$10^{6.00\pm0.42}$
			Viral loads in shrimp (Inf./Tot)	U.D.<br>(0/6)	U.D.<br>(0/6)	[$10^{2.78}$<br>[$10^{2.55}$–$10^{2.94}$]<br>(4/6)	$10^{4.22}$<br>[$10^{2.80}$–$10^{6.36}$]<br>(6/6)	$10^{3.39}$<br>[$10^{2.92}$–$10^{4.92}$]<br>(6/6)	$10^{3.01}$<br>[$10^{2.82}$–$10^{3.23}$]<br>(6/6)
			Severity grades of shrimp	–	–	G0−G1	G0−G3	G0−G3	G0−G3
		Low density ($70/m^2$)	Viral loads in seawater	$10^{4.01\pm0.33}$	$10^{5.98\pm0.26}$	$10^{6.72\pm0.19}$	$10^{6.86\pm0.55}$	$10^{6.20\pm0.55}$	$10^{5.91\pm0.46}$
			Viral loads in shrimp (Inf./Tot)	U.D.<br>(0/6)	U.D.<br>(0/6)	U.D.<br>(0/6)	$10^{4.78}$<br>[$10^{3.65}$–$10^{5.90}$]<br>(2/6)	$10^{3.82}$<br>[$10^{3.27}$–$10^{4.87}$]<br>(4/6)	$10^{3.03}$<br>[$10^{2.64}$–$10^{3.67}$]<br>(4/6)
			Severity grades of shrimp	–	–	–	G0−G3	G0−G3	G0−G2

[1] WSSV genome copies/shrimp.; [2] Viral loads in seawater is presented as mean ± standard deviation (SD) WSSV genome copies/L.; [3] Viral loads in shrimp is presented as median (interquartile range; IQR) WSSV genome copies/mg.; [4] WSSV severity grades were determined based on the WSSV genome copies in shrimp (Kim et al., 2023).; [5] U.D., undetermined.; [6] N.D., Not done.

Table S5. (*Continued*)

Temperature	Injection dose (Donor) [1]	Stocking density	WSSV dynamics	Days (Recipient)					
				1	3	5	7	9	11
30°C	10^3	High density (210/m²)	Viral loads in seawater [2]	$10^{2.78±0.19}$	$10^{5.11±0.19}$	$10^{6.95±0.38}$	$10^{7.04±0.29}$	$10^{7.39±0.11}$	$10^{6.41±0.18}$
			Viral loads in shrimp [3] (Inf./Tot.)	U.D. [5] (0/6)	U.D. (0/6)	$10^{2.12}$ (1/6)	$10^{3.55}$ [$10^{3.00}$−$10^{4.50}$] (6/6)	$10^{7.52}$ [$10^{6.18}$−$10^{7.58}$] (5/6)	$10^{4.65}$ [$10^{4.19}$−$10^{5.09}$] (3/6)
			Severity grades of shrimp [4]	–	–	G0	G0−G3	G0−G3	G0−G3
		Medium density (140/m²)	Viral loads in seawater	U.D.	$10^{4.62±0.40}$	$10^{5.82±0.39}$	$10^{6.73±0.15}$	$10^{7.31±0.22}$	$10^{6.64±0.33}$
			Viral loads in shrimp (Inf./Tot)	U.D. (0/6)	U.D. (0/6)	U.D. (0/6)	U.D. (0/6)	$10^{2.99}$ [$10^{2.67}$−$10^{3.63}$] (4/6)	$10^{3.05}$ [$10^{2.46}$−$10^{4.23}$] (4/6)
			Severity grades of shrimp	–	–	–	–	G0−G2	G0−G3
		Low density (70/m²)	Viral loads in seawater	U.D.	$10^{3.92±0.48}$	$10^{5.53±0.18}$	$10^{5.95±0.62}$	$10^{6.01±0.96}$	$10^{5.38±1.41}$
			Viral loads in shrimp (Inf./Tot)	U.D. (0/6)	U.D. (0/6)	U.D. (0/6)	U.D. (0/6)	$10^{2.48}$ (1/6)	$10^{2.44}$ [$10^{2.44}$−$10^{4.58}$] (3/6)
			Severity grades of shrimp	–	–	–	–	G0	G0−G3

[1] WSSV genome copies/shrimp.; [2] Viral loads in seawater is presented as mean ± standard deviation (SD) WSSV genome copies/L.; [3] Viral loads in shrimp is presented as median (interquartile range; IQR) WSSV genome copies/mg.; [4] WSSV severity grades were determined based on the WSSV genome copies in shrimp (Kim et al., 2023).; [5] U.D., undetermined.; [6] N.D., Not done.

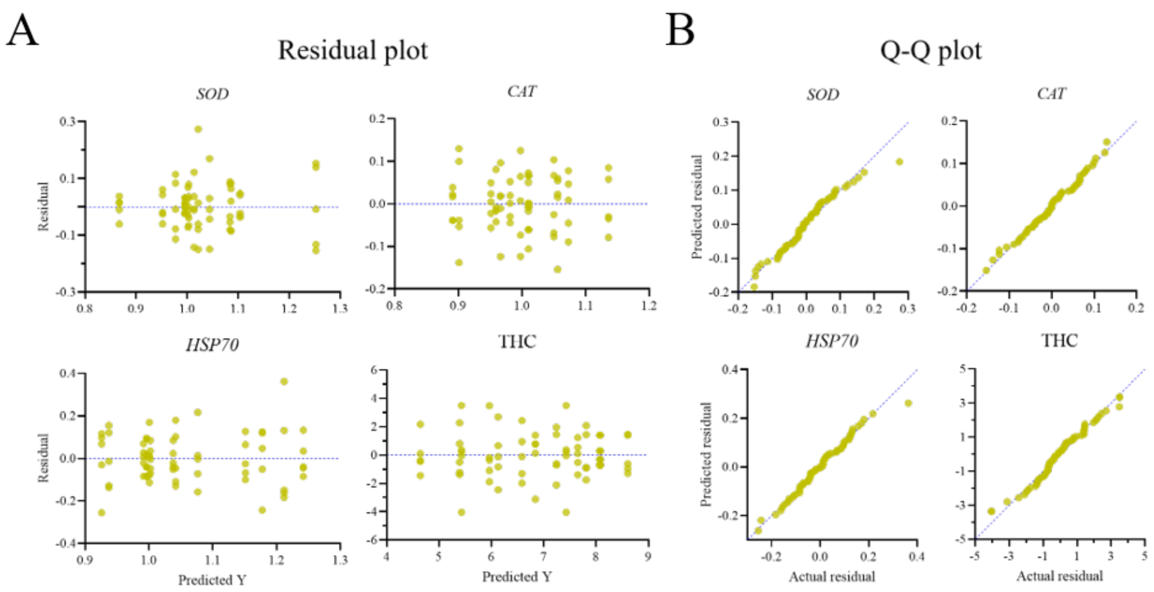

Figure S1. Diagnostic plots of the two-way ANOVA models used in Experiment 1. (**A**) Residual plots and (**B**) Q-Q plots were generated to assess model assumptions for THC and the relative expression levels of *SOD*, *CAT*, and *HSP70*. Residual plots were used to evaluate homogeneity of variance and overall model adequacy, whereas Q-Q plots were used to assess residual normality. No apparent systematic patterns or marked heteroscedasticity were observed in the residual plots, and the Q-Q plots indicated that the residuals were approximately normally distributed. In the residual plots, the blue dashed line represents zero residual. In the Q-Q plots, the blue diagonal line represents the expected distribution under normality. Yellow points indicate individual residuals from each two-way ANOVA model. THC, total hemocyte count; *SOD*, superoxide dismutase; *CAT*, catalase; *HSP70*, heat shock protein 70; ANOVA, analysis of variance.
